# Transcription factor MrpC binds to promoter regions of hundreds of developmentally-regulated genes in *Myxococcus xanthus*

**DOI:** 10.1186/1471-2164-15-1123

**Published:** 2014-12-16

**Authors:** Mark Robinson, Bongjun Son, David Kroos, Lee Kroos

**Affiliations:** Department of Biochemistry and Molecular Biology, Michigan State University, East Lansing, MI 48824 USA

**Keywords:** MrpC, *Myxococcus xanthus*, ChIP-seq, FruA, Fruiting body, Sporulation, Gene regulation, Transcription factor, Cooperative DNA binding, Protein kinase

## Abstract

**Background:**

*Myxococcus xanthus* is a bacterium that undergoes multicellular development when starved. Cells move to aggregation centers and form fruiting bodies in which cells differentiate into dormant spores. MrpC appears to directly activate transcription of *fruA*, which also codes for a transcription factor. Both MrpC and FruA are crucial for aggregation and sporulation. The two proteins bind cooperatively in promoter regions of some developmental genes.

**Results:**

Chromatin immunoprecipitation followed by DNA sequencing (ChIP-seq) and bioinformatic analysis of cells that had formed nascent fruiting bodies revealed 1608 putative MrpC binding sites. These sites included several known to bind MrpC and they were preferentially distributed in likely promoter regions, especially those of genes up-regulated during development. The up-regulated genes include 22 coding for protein kinases. Some of these are known to be directly involved in fruiting body formation and several negatively regulate MrpC accumulation. Our results also implicate MrpC as a direct activator or repressor of genes coding for several transcription factors known to be important for development, for a major spore protein and several proteins important for spore formation, for proteins involved in extracellular A- and C-signaling, and intracellular ppGpp-signaling during development, and for proteins that control the fate of other proteins or play a role in motility. We found that the putative MrpC binding sites revealed by ChIP-seq are enriched for DNA sequences that strongly resemble a consensus sequence for MrpC binding proposed previously. MrpC2, an N-terminally truncated form of MrpC, bound to DNA sequences matching the consensus in all 11 cases tested. Using longer DNA segments containing 15 of the putative MrpC binding sites from our ChIP-seq analysis as probes in electrophoretic mobility shift assays, evidence for one or more MrpC2 binding site was observed in all cases and evidence for cooperative binding of MrpC2 and FruA was seen in 13 cases.

**Conclusions:**

We conclude that MrpC and MrpC2 bind to promoter regions of hundreds of developmentally-regulated genes in *M. xanthus*, in many cases cooperatively with FruA. This binding very likely up-regulates protein kinases, and up- or down-regulates other proteins that profoundly influence the developmental process.

**Electronic supplementary material:**

The online version of this article (doi:10.1186/1471-2164-15-1123) contains supplementary material, which is available to authorized users.

## Background

*Myxococcus xanthus* is a Gram-negative bacterium that provides an attractive model for investigating signaling and gene regulatory mechanisms during a multicellular developmental process [1]. In the soil, rod-shaped *M. xanthus* cells coordinate their movements much like a pack of wolves, allowing them to efficiently lyse prey bacteria and feed on their contents [2]. When the food supply dwindles, cells change their pattern of movements, forming aggregation centers where cells pile on top of one another. The resulting mounds mature into fruiting bodies as some of the cells differentiate into ovoid spores that are resistant to environmental insults and are metabolically quiescent. Other cells remain outside of fruiting bodies as peripheral rods [3, 4] and the majority of cells lyse during the developmental process [5–8]. Signaling between and within cells coordinates their movements, gene expression, and differentiation to reliably build fruiting bodies that each contain about 10^5^ spores [9]. The spores can germinate when nutrients reappear, producing a swarm of rod-shaped cells that can feed efficiently. Here, we focus on the role of a key transcription factor in the *M. xanthus* developmental process, reporting for the first time for this organism the results of genome-wide binding analysis.

The genome of *M. xanthus* is large (9.14 Mb) for a bacterium and it abounds with genes coding for proteins involved in signal transduction and transcriptional regulation [10]. Gene duplication and divergence appears to account for most of the genome expansion. Certain types of genes are overrepresented among those duplicated. For example, many of the 99 predicted serine/threonine protein kinase (STPK) genes [11] appear to have arisen by duplication and divergence [10]. At least 30 of the STPKs play important roles in development, based on gene knockout studies [12]. Likewise, many of the 53 predicted enhancer binding protein (EBP) genes that code for activators of σ^54^ RNA polymerase appear to have arisen in a similar fashion [10] and also are important for development [13]. In addition, there are 137 predicted histidine protein kinase (HPK) genes, in most cases paired with a response regulator (RR) gene, presumably forming a two-component signal transduction system [10]. The tremendous sensory and gene regulatory complexity of *M. xanthus* is proposed to have evolved to support its sophisticated multicellular lifestyle.

Current knowledge of the signaling and gene regulatory network governing *M. xanthus* development has been described in terms of modular design [9, 13]. Starvation, intracellular ppGpp, and extracellular A- and C-signals provide input into three gene regulatory modules designated the EBP cascade, Mrp, and FruA. A simplified view of the regulatory network is shown in Additional file 1. Starvation initiates ppGpp signaling [14, 15] and the EBP cascade [16] and Mrp modules [17, 18]. The EBP cascade module enhances ppGpp signaling [19, 20] and the Mrp module [13]. ppGpp signaling [21, 22] and the EBP cascade module [23] promote production of the A-signal, which is a mixture of amino acids and peptides released by activity of extracellular proteases [24, 25]. A-signaling has been proposed to play a quorum-sensing role that at a high enough cell density stimulates expression of certain genes [26] and causes cells to begin building mounds [27]. The output of the Mrp module is MrpC and its N-terminally truncated form MrpC2, which are transcription factors [28–30] that together with ppGpp signaling [31, 32], the EBP cascade module [33], and proteins involved in A-signal production [34], promote C-signal production. The C-signal appears to be an N-terminally truncated form of the CsgA protein that is produced by proteolytic activity at the cell surface [35–38]. Efficient C-signaling requires alignment of cells, which occurs during fruiting body formation [39–41]. C-signaling [42] and the EBP cascade module [43] positively regulate the activity of the transcription factor FruA by unknown mechanisms that act post-transcriptionally. Another input into the FruA module occurs at the transcriptional level; MrpC and MrpC2 bind to the *fruA* promoter region [44] and appear to directly activate transcription [30]. FruA and MrpC2 have been shown to bind cooperatively to the promoter regions of several genes or operons that are important for aggregation and sporulation [45–48]. Hence, the outputs of the Mrp and FruA modules appear to directly activate transcription of genes crucial for fruiting body formation (Additional file 1).

The Mrp module has been studied in considerable detail. The *mrp* locus was identified by analysis of a transposon insertion mutant with defects in aggregation and sporulation [28]. The locus contains three genes (Additional file 2). Two of the genes are co-transcribed, *mrpA* coding for a predicted HPK and *mrpB* encoding a predicted EBP with a receiver domain that may be the target of MrpA kinase and/or phosphatase activity [28]. The third gene is *mrpC*, which is predicted to code for a transcription factor similar to the cyclic AMP receptor protein (CRP) family [28]. MrpB is necessary for transcription of *mrpC* and MrpC positively autoregulates [28]. MrpC accumulation is also regulated post-transcriptionally. A cascade of two STPKs in which Pkn8 phosphorylates Pkn14, and Pkn14 phosphorylates MrpC, inhibits the accumulation and activity of MrpC during growth [18], since MrpC-P binds DNA poorly [44]. The Esp signaling system also inhibits accumulation of MrpC by stimulating its proteolytic turnover [17, 49]. The finding that MrpC2 is not produced in a *bsgA* mutant suggested that BsgA, a protease in the Lon family [50, 51], might proteolytically process MrpC to MrpC2, although it remains possible that MrpC2 results from alternative initiation of translation [44]. MrpC2 was proposed to be important for developmental progression since it cannot be phosphorylated by Pkn14 and it exhibited higher binding activity than MrpC to the *mrpC* and *fruA* promoter regions [44]. However, we did not detect a difference in the binding of MrpC and MrpC2 to the *fruA* promoter region (reported herein). Since the precise roles of MrpC and MrpC2 remain to be defined and since MrpC is more abundant than MrpC2 in developing cells, we refer to both forms of the protein collectively as MrpC hereafter unless specified otherwise. MrpC governs the timing of development. Premature accumulation of MrpC results in premature accumulation of FruA and premature aggregation and sporulation [17, 18].

How pervasive is regulation by MrpC and FruA during *M. xanthus* development? To begin to address this question, we identified putative MrpC binding sites genome-wide, analyzed their distribution, including their proximity to developmentally-regulated promoters, identified a consensus sequence for MrpC binding, and verified binding of MrpC2 to DNA sequences matching the consensus. We also tested binding of MrpC2 alone or in combination with FruA to longer DNA segments containing putative MrpC binding sites. Our results implicate MrpC as a direct regulator of numerous genes involved in *M. xanthus* development and suggest that cooperative binding of MrpC and FruA is widespread in the *M. xanthus* genome.

## Results

### ChIP-seq reveals a large number of putative MrpC binding sites

*M. xanthus* that had formed nascent fruiting bodies after 18 h of development were subjected to ChIP with antibodies against MrpC. Successful enrichment of MrpC-bound DNA fragments by ChIP was confirmed by ChIP-PCR of the *fmgA* promoter region, which was shown previously to bind MrpC [46]. ChIP-seq generated 8-9 million reads from each of two samples. After alignment of the ChIP-seq reads with the *M. xanthus* genome [10], the alignments were analyzed with QuEST, a statistical tool that has been shown to detect, with high accuracy and positional resolution, genomic regions associated with ChIP-seq peaks of significant enrichment compared with a control [52]. The control data was obtained by performing ChIP-seq with IgG from a non-immunized rabbit. The two ChIP-seq samples with anti-MrpC IgG provided extremely deep sequencing coverage (about 36X for each sample), resulting for each sample in the detection of a similar, large number of peaks with an extremely low estimated false-positive rate (Table 1).

**Table 1:** **Number of significant peaks in each ChIP-seq replicate and false-positive rates**

Sample	Peaks	False-positive rate
1	2132	0.7%
2	2079	0.05%

**Figure 1:**
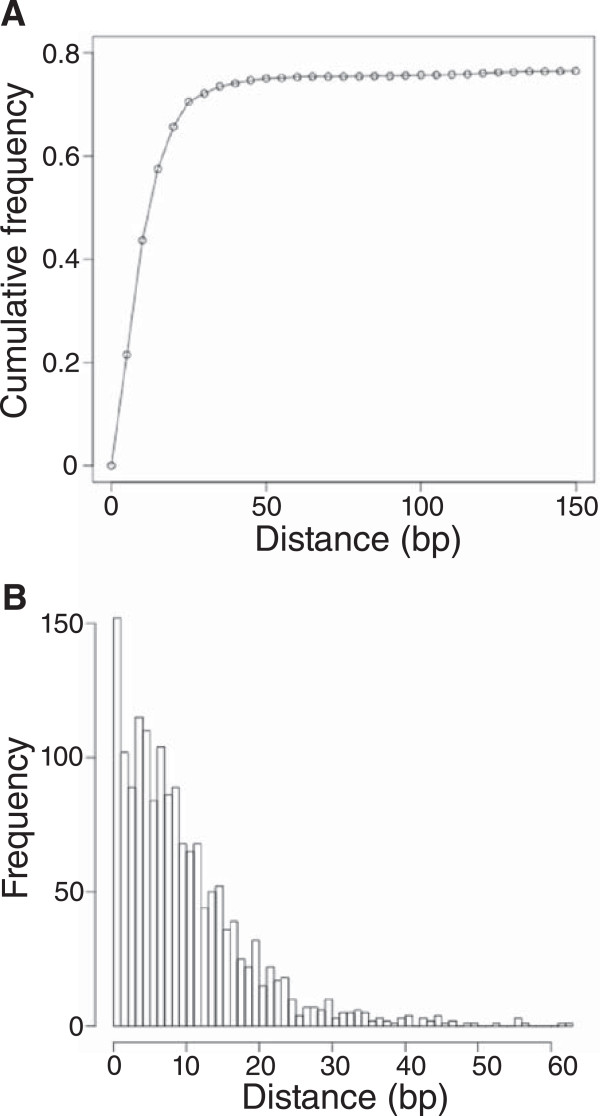
**Distance between replicate peaks. (A)** Cumulative distribution of the distances between nearest replicate peaks. The distance between each peak and the nearest peak in the replicate dataset was first calculated. The proportion of the dataset with a matching peak found at or closer than the selected distances was subsequently plotted. The sharp decrease in the slope of the distribution at about 30 bp indicates the point where increasing the distance threshold includes a diminishing number of new peaks. **(B)** Positional conservation across replicates for 1608 peaks. Frequency histogram generated from the absolute value of the distance between each peak and its closest counterpart in the replicate dataset.

ChIP-seq peaks from the two samples with anti-MrpC were highly reproducible. The cumulative distribution of the distances between nearest replicate peaks shows that approximately 73% of significantly-enriched peaks were found within 30 bp of a significantly-enriched peak in the replicate experiment (Figure 1A). The remaining 27% of peaks were scattered widely at distances of up to 21 kbp from the nearest replicate peak (Figure 1A and data not shown). Making the conservative assumption that replicate peaks separated by more than 65 bp have an increased likelihood of being spurious, we filtered the peaks from the two samples using this cutoff, resulting in 1608 high-confidence peaks (Additional file 3). These peaks exhibit high positional conservation across replicates; the median distance between replicate peaks is 8 bp (Figure 1B). We conclude that MrpC is specifically associated with a large number of sites in the *M. xanthus* genome at 18 h into development, when fruiting bodies have formed, and we refer to these sites as putative MrpC binding sites.

### Known targets of MrpC are found among the sites identified by ChIP-seq analysis

Direct regulatory targets of MrpC have been characterized previously using electrophoretic mobility shift assays (EMSAs), DNase I footprinting, site-directed mutagenesis/reporter gene expression, and ChIP-PCR. As expected, we found known targets of MrpC among the sites identified by our ChIP-seq analysis (Table 2). For *mrpC* [44], *fruA* [30], a site within the *dev* operon [53], and *fmgD* [45], the position of a significantly-enriched ChIP-seq peak (Additional file 3) matched the position of one or more previously-characterized binding sites for MrpC and/or MrpC2. For *fmgBC* [47] and a site upstream of the *dev* operon [53] a ChIP-seq peak matched the position of MrpC2 binding *in vitro*, but the peak was not considered significantly enriched in one of the samples and therefore did not pass our stringency thresholds for inclusion among the 1608 high-confidence peaks (Table 2). For *fmgA*, a ChIP-seq peak was centered approximately 200 bp upstream of the MrpC2 binding site mapped by DNase I footprinting, which was centered at -61.5 relative to the *fmgA* transcriptional start site [46]. The ChIP-seq peak is consistent with a prediction of several closely-spaced MrpC binding sites based on sequence analysis [44]. These predicted MrpC binding sites might be involved in regulation of MXAN_2883, which lies upstream of *fmgA* in divergent orientation. The sites are not involved in *fmgA* regulation based on 5’ deletion analysis of the promoter region fused to a *lacZ* reporter [54]. No significantly-enriched ChIP-seq peaks were found in the *fmgE* promoter region, despite evidence for 3 MrpC2 binding sites in this region [48]. The absence of a significantly-enriched ChIP-seq peak in one or both samples matching the position of previously-characterized binding sites for MrpC and/or MrpC2 in about half the cases represent false-negative results in the ChIP-seq analysis. This suggests that the number of MrpC binding sites in the *M. xanthus* genome at 18 h into development is larger than the 1608 sites we have chosen to analyze. The 1608 sites may be relatively high-affinity sites for binding of MrpC and/or clusters of binding sites (see below and the Discussion). The *fdgA* promoter region from -100 to +1 was not bound by His_10_-MrpC2 in EMSAs (data not shown) and no significantly-enriched ChIP-seq peak was observed in this region (Table 2), providing an example of a true-negative result.

**Table 2:** **Comparison of known MrpC targets with ChIP-seq analysis**

Gene	MXAN	MrpC target	ChIP-seq peak (rank ^a^)	Reference
*mrpC*	5125	Yes	Yes (6)	[44]
*fruA*	3117	Yes	Yes (14)^b^	[30, 44]
*dev*	7265	Yes	Yes (534)	Unpublished data^d^
*fmgD*	1501	Yes	Yes (1358)	[45]
*fmgBC*	4126	Yes	Yes/No^c^	[47]
*dev*	7266	Yes	Yes/No	[53]
*fmgA*	2884	Yes	No	[46]
*fmgE*	3464	Yes	No	[48]
*fdgA*	3225	No	No	[55]

^a^For genes exhibiting a significantly-enriched ChIP-seq peak in both samples, the peak rank among 1608 peaks in Additional file 3 is given in parentheses.

^b^The ChIP-seq peak matched the position of previously-characterized binding sites for MrpC2 that were shown to be important for *fruA* expression [30], although the ChIP-seq peak was closer to the predicted translation start codon of MXAN_3116 (Additional file 3), which is upstream of *fruA* in divergent orientation.

^c^A significantly-enriched peak was observed in one of the two samples subjected to ChIP-seq analysis.

^d^A. Campbell, L. Kroos.

It is instructive to look at the ChIP-seq peak in the *mrpC* promoter region (Additional file 4). There is experimental evidence for at least 6 MrpC binding sites between -204 and -27 relative to the transcriptional start site [44] (between -261 and -84 relative to the translation start codon), yet QuEST analysis of the ChIP-seq data produced a single, broad peak in the region in each replicate (Additional file 4), which on average was centered at -247 relative to the translation start codon (TSC) (Additional file 3). This leads to an important caveat when trying to extrapolate individual binding sites from ChIP-seq peaks. While the reproducibility of the peaks across samples indicated high positional conservation (Figure 1B), individual binding sites that are located in close proximity to each other are not resolved, although they might be recognizable based on similarity to a consensus binding sequence (see below). For comparison, Additional file 4 also shows the ChIP-seq peak in the *fruA* promoter region, where 2 MrpC binding sites have been mapped *in vitro* [30]. The peak is not as high and not as broad as the peak in the *mrpC* promoter region, yet both peaks ranked highly among the 1608 peaks (Additional file 3 and summarized in Table 2), indicative of high-affinity sites for binding of MrpC and/or clusters of binding sites (see below and the Discussion).

### Putative MrpC binding sites are found preferentially in predicted non-coding regions and close to predicted translation start codons

**Figure 2:**
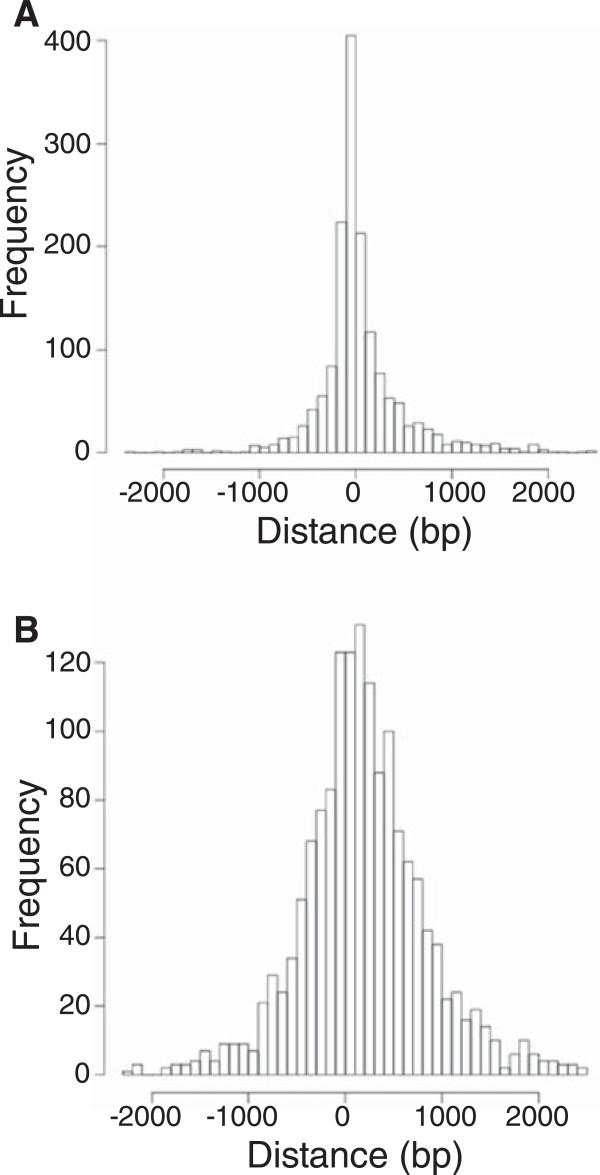
**Distribution of the 1608 putative MrpC binding sites relative to the nearest predicted TSC. (A)** Distances from the ChIP-seq peaks to predicted TSCs. The genomic coordinate of each peak and that of its closest partner in the replicate dataset were averaged, and the distance between this average coordinate and the nearest predicted TSC was then calculated. The resulting distances are plotted as a frequency histogram. **(B)** Distances from randomized peaks to predicted TSCs. Monte Carlo simulation was used to generate a matching number of randomized locations within the genome for comparison with the ChIP-seq dataset. The distance between each randomized location and the nearest predicted TSC was then calculated, and the resulting distances are plotted as a frequency histogram.

A transcription factor such as MrpC would be expected to bind preferentially in non-coding genomic regions. To determine whether the putative MrpC binding sites meet this expectation, the sites were mapped with respect to predicted coding regions in the genome [10]. As can be seen in Table 3, the 1608 ChIP-seq peaks fall preferentially in predicted non-coding regions in comparison with randomly placed peaks within the genome. The ChIP-seq peak distribution is significantly different from the randomized dataset, p < 0.0001 (Fisher’s exact test) [56]. As expected, more than 90% of the randomly placed peaks were in coding regions (Table 3), since more than 90% of the genome consists of coding regions [10]. In contrast, only 61% of the ChIP-seq peaks were in coding regions (Table 3).The putative MrpC binding sites also differed from the randomly located sites in terms of distance to the nearest predicted TSC. The putative MrpC binding sites were narrowly distributed around a maximum immediately upstream of the nearest predicted TSC (Figure 2A), as would be expected for sites involved in gene regulation. The randomly chosen sites were broadly distributed relative to the nearest predicted TSC (Figure 2B).

**Table 3:** **Distribution of ChIP-seq peaks across non-coding and coding regions relative to a randomized dataset**

	Non-coding region	Coding region
ChIP-seq peaks	626	982
Random sites	149	1459

### Functional annotation of genes near putative MrpC binding sites reveals no over-represented categories of genes

Additional file 3 lists the distance between each putative MrpC binding site and the nearest TSC, as well as the functional annotation of the corresponding gene. Since most functional transcription factor binding sites are located close to a TSC in bacteria, any putative MrpC binding sites not located between 400 bp upstream and 100 bp downstream of a predicted TSC [10] were removed from the dataset. Of the 1059 remaining sites, 334 were associated with divergent genes. In these cases, both genes (hence 1393 genes total) were included in the analyses that follow.

The distribution of the distance from each putative MrpC binding site to the nearest predicted TSC (or the two nearest predicted TSCs for sites associated with divergent genes) was plotted. The putative MrpC binding sites were strongly skewed toward the region immediately upstream of a predicted TSC as compared with an equal number of sites placed at random in the genome using a Monte Carlo simulation (Additional file 5). These results suggest that many of the putative MrpC binding sites are biologically relevant sites involved in gene regulation.

Comparing the functional categories of the 1393 genes with that of all genes in the genome, no categories are over-represented and only one is under-represented with high statistical confidence (Table 4). The under-represented category is disrupted reading frames, which are presumably non-functional genes, so it is neither surprising that these are under-represented nor particularly informative. The classes of genes that may be over-represented, although not with high statistical confidence, include most notably genes in the categories of protein fate, regulatory functions, signal transduction, and transcription. This suggests that MrpC is regulating other regulators in the network governing developmental gene expression.

**Table 4:** **Functional categories of genes associated with putative MrpC binding sites and the statistical significance of the categorical distributions relative to the whole genome**

Functional category ^a^	Genome-wide	Putative MrpC sites	Expected ^b^	P-value ^c^
Amino acid biosynthesis	74	11	14	0.69
Biosynthesis of cofactors, prosthetic groups, and carriers	115	20	22	0.88
Cell envelope	699	140	132	0.66
Cellular processes	249	52	47	0.68
Central intermediary metabolism	52	12	10	0.83
Disrupted reading frames	49	0	9	0.002
Disrupted reading frames: Mobile and extrachromosomal element functions	4	0	1	1.00
DNA metabolism	102	17	19	0.87
Energy metabolism	320	67	60	0.59
Fatty acid and phospholipid metabolism	134	15	25	0.15
Hypothetical proteins	2812	552	531	0.44
Mobile and extrachromosomal element functions	90	9	17	0.17
Protein fate	296	65	56	0.46
Protein synthesis	162	29	31	0.90
Purines, pyrimidines, nucleosides, and nucleotides	58	6	11	0.33
Regulatory functions	286	66	54	0.30
Signal transduction	259	62	49	0.25
Transcription	99	28	19	0.24
Transport and binding proteins	404	59	76	0.16
Unknown function	1115	183	210	0.15
Total	7379	1393	1393	

^a^Functional categories were assigned by the J. Craig Venter Institute and amended as described previously [57].

^b^The number expected was calculated by multiplying the number of genes in a functional category genome-wide by the ratio of putative MrpC binding sites near (i.e., between -400 and +100) the predicted start codon of a gene (i.e., 1393) to the total number of genes in the genome that have been assigned a functional category (i.e., 7379) [57].

^c^The P-value associated with the difference between the number of putative MrpC sites observed versus the number expected was calculated using Fisher’s exact test [56].

### Putative MrpC binding sites are preferentially located near the 5’ ends of developmental genes

As described above, MrpC and MrpC2 appear to directly regulate several developmental genes (Table 2). To extend this analysis, we compared the 1393 genes whose predicted TSC is near (i.e., between -400 and +100) a putative MrpC binding site, to various lists of developmental genes. Lists of genes directly involved in fruiting body formation, or significantly down- or up-regulated during development, have been described previously [58]. We found that putative MrpC binding sites are over-represented near developmentally up-regulated genes with high statistical confidence (Table 5). They may be over-represented near genes directly involved in development and near genes down-regulated during development, although not with high statistical confidence. In addition to the lists described previously [58], we generated a list of “potentially interesting” genes involved in motility, signaling, or gene expression that may play a role in development (Additional file 6). Putative MrpC binding sites were not found to be over-represented near the potentially interesting genes in general, but they may be over-represented near the promoter regions of these genes when their likely operon organization is take into account (Table 5). The genes from each of the four lists that are near a putative MrpC binding site are summarized in Additional file 7. These genes are candidates for direct regulation by MrpC and MrpC2.

**Table 5:** **Developmental genes associated with putative MrpC binding sites and the statistical significance relative to all genes**

List ^a^	Number of listed genes	Putative MrpC sites	Expected ^b^	P-value ^c^
Directly involved	95	27	18	0.17
Down-regulated	424	96	80	0.20
Up-regulated	410	133	77	<0.0001
Potentially interesting	345	63	65	1.0
Potentially interesting promoter regions	207	54	39	0.10

^a^Genes directly involved in fruiting body formation, down-regulated, or up-regulated are from Tables S3, S5, and S6, respectively, of Huntley et al. [58]. Potentially interesting genes are listed in Additional file 6. Potentially interesting promoter regions does not include genes likely to be in operons that are downstream of the first gene.

^b^The number expected was calculated by multiplying the number of listed genes by the ratio of putative MrpC binding sites near (i.e., between -400 and +100) the predicted start codon of a gene (i.e., 1393) to the total number of genes in the genome that have been assigned a functional category (i.e., 7379) [57].

^c^The P-value associated with the difference between the number of putative MrpC sites observed versus the number expected was calculated using Fisher’s exact test [56].

Our finding that developmentally up-regulated genes are over-represented near putative MrpC binding sites (Table 5) is consistent with MrpC functioning as an activator of transcription. It is important to note that the list of up-regulated genes used in this analysis [58] is incomplete. For example, only 3 of the 9 genes in Table 2 are on the list, yet all 9 are up-regulated during development based on *lacZ* fusion and/or RT-PCR analysis. The list is derived from DNA microarray experiments with RNA harvested at various times between 0 and 24 h into development [59], so up-regulated genes could have been missed for several reasons (e.g., microarray experiments may not be as sensitive as *lacZ* fusion analysis for genes that are weakly regulated or produce unstable mRNA, and some genes may be induced later than 24 h poststarvation). Nevertheless, genes that are up-regulated during development based on the microarray experiments and near a putative MrpC binding site based on our ChIP-seq analysis include 9 predicted to code for an HPK (MXAN_0736, 0928, 0931, 1014, 1553, 3036, 3290, 5628, 7002), 4 for an RR (MXAN_0524, 6012, 7001, 7024), 2 for a hybrid HPK/RR (MXAN_6315, 6734), 4 for an STPK (MXAN_0724, 0930, 1710, 2680), 4 for a DNA-binding protein (MXAN_0228, 2913, 3089, 4446), and 3 for a σ factor (MXAN_0947, 5101, 6209) (Additional file 7, column C). Therefore, MrpC very likely up-regulates more than 24 regulators in the network governing developmental gene expression.

Among the genes mentioned above that are up-regulated during development based on microarray experiments and near a putative MrpC binding site, several are known to be directly involved in fruiting body formation (Additional file 7, column A). These include the HPKs *espA* [60], *sdeK* [61], *hpk8* [59], the STPKs *pktA5* [62], *pkn8* [18], *pskA12* [12], and the σ factor *sigC* [63, 64]. These seven genes are highlighted yellow in columns A and C of Additional file 7.

Twenty other genes that are known to be directly involved in fruiting body formation were found to be near a putative MrpC binding site (Additional file 7, column A). Eleven of these are up-regulated during development, based on *lacZ* fusion and/or RT-PCR analysis (see below for references), even though they were not detected as up-regulated in the DNA microarray experiments [58, 59]. In two cases, *prw* [65] and *espC* [49], only expression at the protein level has been examined (by immunoblot), and it was observed to increase during development. Expression of *pkn13*, *pktE2*, *crdA*, *pktD6*, and *pktA1* has not been reported, expression of *sasS-lacZ* shows little change but was only measured during the first 8 h of development [66], and the level of *hthA* transcript decreased strongly by 6 h into development [67]. Hence, at least 13 genes directly involved in development and near a putative MrpC binding site are up-regulated during development, based on methods other than microarrays. These genes are also highlighted yellow in column A of Additional file 7. Several of these genes code for protein kinases (PKs), including the HPKs *hpk37* [59], *mrpA* [28], *espC* [68], *asgD* [69] and the STPKs *pkn9* [70], *pkn1* [71], *pkn6* [72]. Adding these 7 to the 9 HPKs, 2 hybrid HPK/RRs, and 4 STPKs mentioned above, a total of 22 PKs are likely up-regulated by MrpC. Some of these negatively regulate development, while others positively regulate development. We conclude that MrpC very likely has a profound effect on phosphorylation-dependent signaling that regulates *M. xanthus* development.

In addition to PKs, putative MrpC binding sites are located near genes that code for transcription factors (*fruA*, *actB*, *mrpC*, *crdA*, *sigC*, *hthA*) and spore proteins (*prw*, *nfsA*, *nfsH*) known to be important for fruiting body formation (Additional file 7, column A). It was mentioned above that *fruA* and *mrpC* were known targets of MrpC (Table 2). Also, *sigC* was noted since it was on the list of genes up-regulated during development based on DNA microarray experiments (Additional file 7, columns A and C). However, dependence of *sigC* expression on MrpC has not been tested, and the putative MrpC binding site near *sigC* is actually closer to the divergent gene MXAN_6208 predicted to code for a hypothetical protein (Additional file 3), although not by much. Since *sigC* but not MXAN_6208 was up-regulated during development in the DNA microarray experiments [58, 59], it is more likely that MrpC activates *sigC* than MXAN_6208 transcription, but this will need to be tested. The *crdA* gene is in divergent orientation relative to *crdB* (MXAN_5152), the first gene of the *che3* chemosensory system cluster [73], and the putative MrpC binding site in this region is closer to the predicted *crdB* TSC (Additional file 3). The putative MrpC binding site near the *prw* gene, which codes for the abundant spore protein W [65], is within the upstream, divergent MXAN_2490 gene (Additional file 3). In addition to divergent genes, operons also need to be considered. For example, *actB* is in the *actABCD* operon [74]. The putative MrpC binding site is located at -353 relative to the predicted *actB* TSC (Additional file 3). Therefore, the putative MrpC binding site is within *actA*, at about +770 relative to the transcription start site of the operon [74]. Whether MrpC directly regulates the promoter of the *act* operon or a suboperonic promoter located within *actA* will require careful analysis, especially since the *act* operon promoter is known to be positively regulated by FruA [33] and MrpC appears to be a direct activator of *fruA* transcription [30]. The *nfsA* and *nfsH* genes are likely in an eight-gene operon [57] whose products are involved in deposition of the polysaccharide spore coat [75]. Two putative MrpC binding sites are located at -331 and -137 relative to the predicted *nfsA* TSC, and one putative MrpC binding site is located within *nfsG* (MXAN_3177) at -292 relative to the predicted *nfsH* TSC (Additional file 3). These examples illustrate complexities of interpreting the likely significance of putative MrpC binding sites for gene regulation. Adding to the uncertainty, most transcription start sites have not been mapped. Therefore, even in simple cases like the *hthA* gene, where a putative MrpC binding site is located at -79 relative to the predicted TSC (Additional file 3), whether MrpC binding to this site accounts for the observed down-regulation of *hthA* [67] during development is unclear.

Ninety-six genes that are down-regulated during development were found to be near putative MrpC binding sites (Additional file 7, column B). MrpC has not been reported to act as a repressor of transcription, although it has been suggested to delay transcription of *fmgD* by competing with FruA for binding to a site from which FruA appears to activate transcription [45]. In striking contrast to the genes that are up-regulated during development and near a putative MrpC binding site, the genes that are down-regulated do not include PKs or RRs. On the other hand, the genes that are down-regulated include 13 in the functional category [57] of protein fate (MXAN_0645, 1176, 1678, 1967, 2016, 2286, 2791, 3012, 3129, 3160, 4692, 4894, 6849), more than twice as many as the 6 up-regulated genes in that category (MXAN_0100, 644, 1501, 2015, 4054, 4547) (Additional file 7, columns B and C). We conclude that MrpC may repress transcription of nearly 100 genes during development and in particular this may resculpt the proteome.

Putative MrpC binding sites were not over-represented near genes we considered “potentially interesting”, but if we take into account that many of these genes are likely co-transcribed in operons, MrpC binding sites may be enriched in the promoter regions of potentially interesting genes (Table 5). Genes likely to be in operons are highlighted in Additional file 6. The number of potentially interesting promoter regions, 207, is considerably less than the total number of potentially interesting genes, 345 (Table 5). Strikingly, in 17 of 26 cases with a putative MrpC binding site near a gene likely in an operon (Additional file 7, column D; Additional file 6), the site is near the predicted TSC of the likely first gene of the operon (highlighted green in Additional file 6), suggesting that the operon promoter is regulated by MrpC. In the other cases, MrpC may regulate a suboperonic promoter.

Some of the potentially interesting genes near a putative MrpC binding site are down- or up-regulated during development based on the DNA microarray experiments [58, 59] (highlighted red or green in Additional file 7, column D). Of the 9 genes down-regulated, 2 are likely the first gene of an operon, one implicated in A motility (*gltB,* MXAN_2539) and the other in E-signaling (*esgA,* MXAN_4564) (Additional file 6). The other 7 are implicated in S motility (*sgmH,* MXAN_2526*; efp,* MXAN_5769*; epsL,* MXAN_7437), A motility (*agmO,* MXAN_2538), gene expression (*ihfA,* MXAN_3596*; rpoD,* MXAN_5204), or spore formation (*sapA,* MXAN_7407). Of the 7 genes up-regulated, 1 is likely the first gene of an operon implicated in spore formation (*sapB,* MXAN_3885). The other 6 are implicated in C-signaling (*popD,* MXAN_0207), phase variation (*xre228,* MXAN_0228), A motility (*cglD,* MXAN_0962*; aglCR,* MXAN_7296), A-signaling (*asgB,* MXAN_2913), or S motility (*sgmK,* MXAN_2922). We conclude that MrpC may both down- and up-regulate particular genes involved in both S motility and A motility, as well as in extracellular signaling and spore formation during development.

Taken together, our results imply that MrpC directly activates or represses hundreds of genes involved in signaling, transcription, spore formation, protein fate, and motility during development.

### Putative MrpC binding sites are enriched for a motif that strongly resembles a consensus sequence for MrpC binding

**Figure 3:**
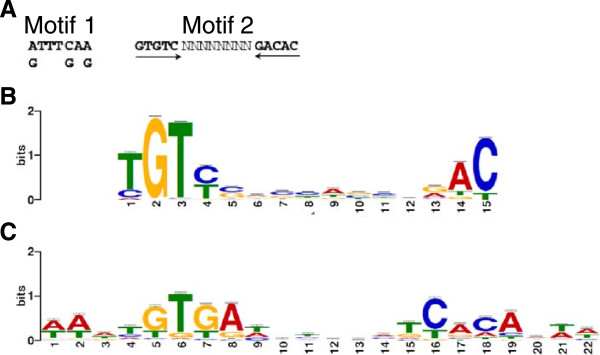
**MrpC binding site consensus sequences and the consensus sequence for binding of** ***E. coli*** **CRP. (A)** MrpC-binding motif 1 is based on 6 sites in the *mrpC* promoter region and motif 2 is based on 2 sites in the *mrpC* promoter region and 2 in the *fruA* promoter region [44]. **(B)** Consensus binding sequence for MrpC based on MEME analysis of the top 300 peaks from ChIP-seq. The height of the letters in the sequence logo represents the information content [77]. **(C)** Consensus binding sequence for *E. coli* CRP displayed as in panel **B**.

Two distinct binding site consensus sequences have been proposed for MrpC (Figure 3A) [44]. To clarify the binding site sequence preference of MrpC, sequences corresponding to the 500 top-ranked putative MrpC binding sites (Additional file 3) were extracted, including 50 bp of flanking sequence on each side of the ChIP-seq peak maxima. MEME, an expectation maximization algorithm [76], was used to discover motifs that are statistically over-represented in these sequences. Only one significant motif was identified (repeatedly) among the various partitions of the data that were searched by MEME (Figure 3B). This motif is a strong match for the larger of the two motifs proposed previously [44] (Figure 3A). The motif is an imperfect palindrome that can be represented as TGTYN_8_RAC, consistent with MrpC binding as a dimer. MrpC is dimeric [18], a common feature of DNA-binding proteins in the CRP family to which MrpC belongs [28].

The motif identified by MEME analysis of putative MrpC binding sites was compared with binding motifs stored in RegTransBase [78] using TOMTOM [76]. A significant match was identified to the consensus binding sequence for the cyclic-AMP receptor protein (CRP) of *E. coli* (Figure 3C). Based on amino acid sequence similarity, MrpC was proposed to be a member of the CRP family [28]. Our data indicate that the two proteins recognize a similar DNA sequence.

### Experimental verification of putative MrpC binding sites

**Figure 4:**
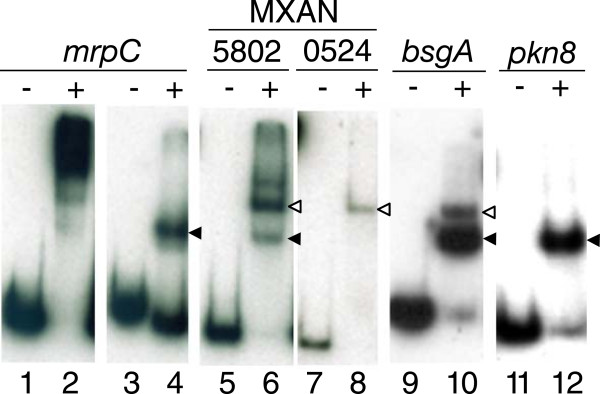
**Binding of MrpC2 to candidate genes from ChIP-seq.** For the indicated genes, approximately 200 bp of DNA surrounding a peak from the ChIP-seq analysis was amplified by PCR with ^32^P-labeled primers. These probes (2 nM) alone (-) or after addition of His_10_-MrpC2 (+) (1 μM, except lane 4 in which 0.5 μM was added) were subjected to EMSAs. MXAN_5802 and 0524 were analyzed on the same gel, with intervening lanes removed, as indicated by narrower separation between panels. Filled and open arrowheads indicate complexes inferred to have one or two His_10_-MrpC2 bound, respectively.

We purified N-terminally His_10_-tagged versions of MrpC and MrpC2 from *E. coli* engineered for overexpression. We found that expression of His_10_-MrpC appeared to be toxic to *E. coli*, but we managed to purify a small amount for comparison with His_10_-MrpC2, which did not appear to be toxic and was therefore easier to obtain. The two proteins were indistinguishable in binding to a region upstream of the *fruA* promoter (Additional file 8). Previously, Nariya and Inouye [44] reported that His_10_-MrpC2 has 8-fold higher binding activity than His_10_-MrpC for the same DNA fragment from the *fruA* upstream region. We do not understand this discrepancy, but since we observed no difference in binding and since His_10_-MrpC2 was much easier to obtain, we used it to test binding to sites identified by our ChIP-seq analysis. We chose putative MrpC binding sites near genes with a variety of characteristics (Additional file 9), amplified 200 bp of DNA surrounding the putative site, and performed EMSAs. As shown previously [44] and as predicted by our ChIP-seq analysis, His_10_-MrpC2 bound a DNA fragment from the *mrpC* upstream region (Figure 4, lanes 1-4). Different concentrations of His_10_-MrpC2 produced complexes with different migration distances, consistent with the presence of multiple binding sites on the DNA fragment. A DNA fragment encompassing a putative MrpC binding site upstream of MXAN_5802 produced 3 shifted complexes (Figure 4, lane 6), suggesting the presence of multiple binding sites, which may account for the high rank of this site on the list of ChIP-seq peaks (Additional file 3). The putative site upstream of MXAN_0524 (also high on the list in Additional file 3) produced a single complex that was inferred to have 2 His_10_-MrpC2 bound based on its migration (Figure 4, lane 8), and 2 sites were subsequently verified (see below). The putative sites upstream of *bsgA* and *pkn8* produced primarily a complex inferred to be bound by 1 His_10_-MrpC2 (Figure 4, lanes 10 and 12), although a small amount of a complex inferred to have 2 His_10_-MrpC2 bound was observed for *bsgA*. All the other putative MrpC binding sites we tested in this way produced one or more complexes (Additional file 10), as summarized in Additional file 9. As noted above, the *fdgA* promoter region from -100 to +1 was not bound by His_10_-MrpC2 in EMSAs (data not shown). We conclude that most sites identified by our ChIP-seq analysis likely could be bound specifically by one or more His_10_-MrpC2 *in vitro*.

**Figure 5:**
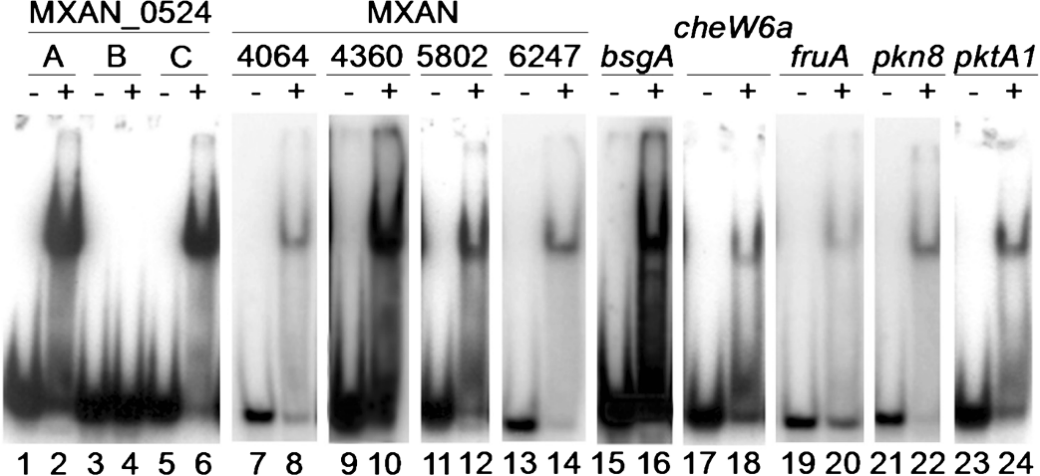
**Binding of MrpC2 to predicted sites.** For the indicated genes, the sequence matching the motif in Figure 3B, plus 11-14 bp on each side, were synthesized and ^32^P-labeled oligonucleotides were annealed. These probes (2 nM) alone (-) or after addition of His_10_-MrpC2 (+) (1 μM) were subjected to EMSAs. MXAN_0524 had two matches to the motif (probes A and C), and probe B contains the sequence in between the matches plus 10 bp of each match (Additional file 12).

We searched for the motif identified by MEME analysis of putative MrpC binding sites (Figure 3B) in the regions encompassing the putative sites tested above. Not counting *mrpC* and *fruA*, which were already known to have matches to the motif [44], we found one or more matches in 9 of the remaining 15 regions (Additional file 9). For each match, we obtained oligonucleotides corresponding to the match plus 11-14 bp on each side and used the annealed oligonucleotides as a probe in EMSAs with His_10_-MrpC2. In each case, a complex was observed (Figure 5). For the region upstream of MXAN_0524, which had two matches to the motif, probes matching each motif formed a complex, but probe B corresponding to the sequence in between the two motifs and including 10 bp of each motif did not form a complex. These results validate the predictive value of the motif for identifying sequences bound specifically by His_10_-MrpC2 *in vitro*.

### Evidence for cooperative binding of MrpC2 and FruA

His_10_-MrpC2 and FruA-His_6_ have been shown to bind cooperatively in the *fmgA* promoter region by DNase I footprinting analysis [46]. In EMSAs, cooperative binding produced a slow-migrating complex more abundantly than could be accounted for by binding of each protein alone [46], and similar results provide evidence for cooperative binding of the two proteins in the *fmgBC* [47], *fmgD* [45], *fmgE* [48], and *dev* [53] promoter regions. Therefore, we used EMSAs to test for evidence of cooperative binding to the regions from our ChIP-seq analysis that were bound by His_10_-MrpC2 as described above. Of 15 regions tested, we could detect binding of FruA-His_6_ alone in 8 cases and evidence for cooperative binding with His_10_-MrpC2 in 13 cases (summarized in Additional file 9).

**Figure 6:**
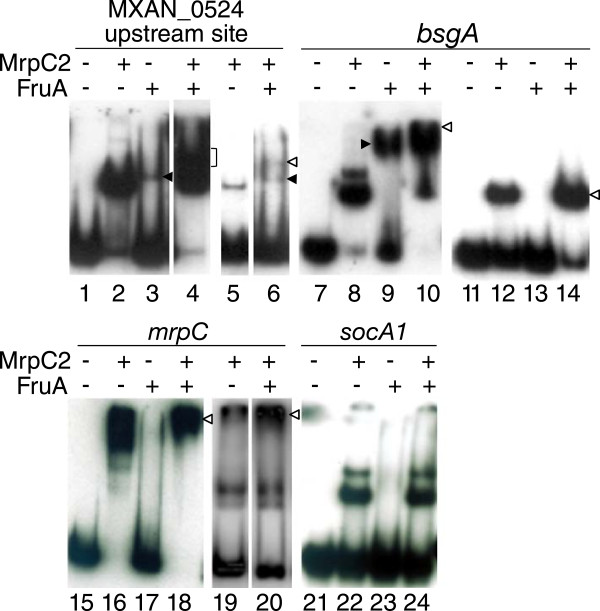
**Patterns of MrpC2 and FruA binding to candidate genes from ChIP-seq.** For the indicated genes, approximately 200 bp of DNA surrounding a peak from the ChIP-seq analysis was amplified by PCR with ^32^P-labeled primers. The MXAN_0524 probe contained only the upstream predicted MrpC2 binding site. The DNA probes (2 nM) were incubated with His_10_-MrpC2 (1 μM) and/or FruA-His_6_ (3 μM) as indicated (unless noted below), and subjected to EMSAs. A lower concentration of His_10_-MrpC2 (0.03 μM in lanes 5 and 6; 0.1 μM in lanes 12, 14, 19, and 20) and FruA-His_6_ (1.5 μM in lanes 13 and 14) was used in some experiments. Filled arrowheads pointing leftward or rightward are inferred to indicate complexes with one or more FruA-His_6_ bound, respectively. Brackets and open arrowheads indicate novel or more abundant complexes produced by the combination of proteins than by either protein alone. Panels with a narrower separation were analyzed on the same gel, with intervening lanes removed.

We observed 4 patterns of FruA-His_6_ and His_10_-MrpC2 binding. Figure 6 shows an example of each pattern. As an example of the first pattern, FruA-His_6_ alone bound weakly to the MXAN_0524 upstream region (Figure 6, lane 3). The combination of FruA-His_6_ and His_10_-MrpC2 produced a slow-migrating complex(es) more abundantly than could be accounted for by binding of each protein alone (Figure 6, lane 4 bracket), providing evidence for cooperative binding. At a much lower concentration of His_10_-MrpC2 alone, a single complex was observed (Figure 6, lane 5, note that this probe has only one of the two sites in this region). This complex was not observed when FruA-His_6_ was added (Figure 6, lane 6). Rather, two complexes were observed, one that co-migrated with the complex produced by FruA-His_6_ alone (black arrowhead) and the other migrating more slowly (white arrowhead), which may be due to cooperative binding of the two proteins. A similar pattern of weak binding by FruA-His_6_ alone, and a slow-migrating complex(es) produced more abundantly by the combination of FruA-His_6_ and His_10_-MrpC2 (only a high concentration was tested) than could be accounted for by binding of each protein alone (i.e., evidence for cooperative binding), was observed for the *pktA1*, MXAN_2902, MXAN_4360, and *mrpA* regions (Additional file 10, top row).

A slightly different pattern was observed for the *bsgA*, MXAN_6247, and *cheW6a* regions (Figure 6; Additional file 10, second row). These upstream regions were bound strongly by FruA-His_6_ alone, producing a complex whose migration distance suggested more than 1 FruA-His_6_ was bound. When His_10_-MrpC2 was added, a slow-migrating complex(es) was produced more abundantly than could be accounted for by binding of each protein alone, providing evidence for cooperative binding. At a 2-fold lower concentration of FruA-His_6_ alone, no complex was observed for the *bsgA* region (Figure 6, lane 13) and a complex suggestive of 1 FruA-His_6_ bound was barely detectable for the MXAN_6247 (Additional file 10, lane 23). Addition of a 10-fold lower concentration of His_10_-MrpC2 to the 2-fold lower concentration of FruA-His_6_ produced slightly more complex than could be accounted for by binding of each protein alone (Figure 6, lane 14; Additional file 10, lane 24), consistent with cooperative binding, but surprisingly a novel, slow-migrating complex was observed only for the MXAN_6247 region.

A third pattern was observed for the *mrpC* upstream region. FruA-His_6_ alone failed to produce a distinct complex (Figure 6, lane 17), but in combination with His_10_-MrpC2, a slow-migrating complex(es) was produced more abundantly than could be accounted for by binding of each protein alone (Figure 6, lane 18), suggestive of cooperative binding. At a 10-fold lower concentration of His_10_-MrpC2, more slow-migrating complex(es) was produced when FruA-His_6_ was added (Figure 6, lanes 19 and 20), consistent with cooperative binding. Likewise, FruA-His_6_ alone failed to produce a distinct complex with the MXAN_5802, *frzS*, *pkn8*, and *rpoE1* regions, but there was evidence of cooperative binding in combination with His_10_-MrpC2 (Additional file 10, third row).

The fourth pattern, failure of FruA-His_6_ alone to bind and lack of cooperative binding, was observed for the regions upstream of *socA1* (Figure 6, lanes 23 and 24) and *pilA* (Additional file 10, lanes 49 and 50).

All together, we found evidence of cooperative binding of MrpC2 and FruA in 13 of 15 regions tested (Additional file 9). Adding this to previous studies that provided evidence for cooperative binding in 5 of 5 regions examined [45–48, 53], it appears that cooperative binding of MrpC2 and FruA is widespread in the *M. xanthus* genome.

## Discussion

Our ChIP-seq analysis, consensus binding site identification, and EMSA experiments provide strong evidence for the conclusion that MrpC and MrpC2 bind to promoter regions of hundreds of developmentally-regulated genes in *M. xanthus*, in many cases cooperatively with FruA. To our knowledge, this is the first report of genome-wide binding analysis for a transcription factor in *M. xanthus*. While it was gratifying to find some of the known targets of MrpC among the sites identified by our ChIP-seq analysis, others were missed, so undoubtedly there remain more MrpC binding sites to be found. Despite missing some of the functional MrpC binding sites, and finding others located far from the nearest promoter region and therefore perhaps not functional for transcriptional regulation, we found a large number of putative MrpC binding sites in promoter regions and we verified binding of MrpC2 *in vitro* in all 15 cases tested, so many of the putative MrpC binding sites very likely regulate transcription. Moreover, in 13 of the 15 cases, and in 5 cases examined previously, MrpC2 appeared to bind cooperatively with FruA to DNA, suggesting that many genes are coordinately regulated by these two transcription factors.

### A large number of putative MrpC binding sites

The number of putative MrpC binding sites revealed by our ChIP-seq analysis was surprisingly large. Typically, genome-wide binding analyses (ChIP-chip or ChIP-seq) have yielded about an order of magnitude lower number of putative binding sites for bacterial transcription factors [79–84]. The *M. xanthus* genome is larger than most bacterial genomes, but it is only about twice as large as those of well-studied model organisms like *E. coli*, *Bacillus subtilis*, and *Caulobacter crescentus*, so genome size can only partly account for the unexpectedly large number of putative MrpC binding sites. Since MrpC was proposed to be a member of the CRP family based on sequence similarity [28] and since MrpC recognizes a similar consensus sequence as *E. coli* CRP (Figure 3), it may be useful to compare genome-wide studies of *E. coli* CRP. Identification of the CRP regulon using *in vitro* and *in vivo* transcriptional profiling revealed 176 up-regulated operons and 16 down-regulated operons [85]. ChIP-chip analysis yielded only 68 high-occupancy sites, but many thousands of weak sites scattered throughout the *E. coli* chromosome [86], possibly related to the >10,000 lower-affinity sites predicted by computational analysis [87]. Follow-up experiments on 11 previously uncharacterized targets identified by the genomic approaches [85, 86] demonstrated CRP binding *in vitro* in 8 cases and transcriptional regulation by CRP *in vivo* in only 5 cases [88]. These results highlight the complexities of genome-wide studies. Each approach, including ChIP-seq [89], has limitations that can generate false positives, and follow-up experiments like EMSAs and transcript analysis have limitations as well. Nevertheless, many of the 1608 putative MrpC binding sites identified by our ChIP-seq analysis are likely to function in transcriptional regulation since 1) they exhibit high positional conservation across two replicates, 2) several known targets of MrpC were found, 3) they are preferentially located in predicted non-coding regions and close to predicted TSCs, 4) those in promoter regions are greatly over-represented near developmentally up-regulated genes and near the first gene of operons we deemed potentially interesting since they are involved in motility, signaling, or gene expression that may play a role in development, 5) all 15 sites we tested were bound by MrpC2 *in vitro*, 6) bioinformatic analysis of the sites identified a consensus sequence that was highly predictive of MrpC2 binding *in vitro* (all 11 cases tested).

Putative MrpC binding sites that are located far from promoter regions are less likely to participate in transcriptional regulation. Of the 1608 putative MrpC binding sites, 549 were not located between -400 and +100 relative to a predicted TSC. Most of these sites are in coding regions. Whether these sites reflect a role of MrpC in organizing the chromosome within the cell, as has been speculated for *E. coli* CRP [86] and *B. subtilis* SpoIIID [81], remains to be seen.

Much more work is needed to explore the functionality of the 1608 putative MrpC binding sites revealed by our ChIP-seq analysis. In addition, there undoubtedly remain more functional MrpC binding sites to be found. Only half of the previously known MrpC binding sites were detected as significant peaks in both replicates of our ChIP-seq analysis (Table 2). The majority of the putative MrpC binding sites we tested, including the 9 most highly ranked, appeared to be bound by more than one MrpC2 (Additional file 9), suggesting that multiple binding sites contributed to detectability in our ChIP-seq analysis. Presumably, the affinity of MrpC for individual sites was another major contributor to detectability. Beyond these issues of detectability, it is very likely that MrpC binding changes during the course of development, so ChIP-seq at times other than 18 h post-starvation would very likely reveal additional MrpC binding sites as well.

### MrpC is implicated as a transcriptional activator of hundreds of developmental genes, especially protein kinases and transcription factors

**Figure 7:**
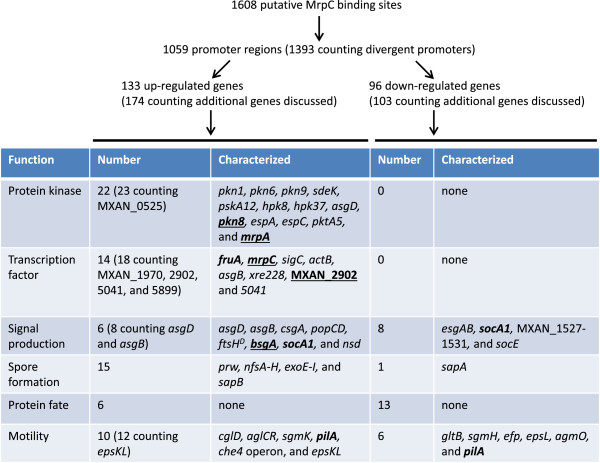
**Analysis of putative MrpC binding sites with respect to up- or down-regulated developmental genes and their functions**. Putative MrpC binding sites are listed in Additional file 3. Up- or down-regulated developmental genes identified in microarray experiments [58, 59] are listed in Additional file 7, and additional genes discussed are described in the text (where references can be found). The table lists the number of up- or down-regulated genes in each functional category and lists those characterized (as described in the text). Genes in bold were bound in their promoter region by His_10_-MrpC2 and genes that appeared to be bound cooperatively by His_10_-MrpC2 and FruA-His_6_ are also underlined (Figures 4, 5 and 6; Additional files 8, 9 and 10; note that *fruA* was not tested for cooperative binding). The *pilA*[90] and *epsL*[91] genes are listed twice because they are first up-regulated and then down-regulated during development. The *socA1* gene is listed twice because it is down-regulated in rod-shaped cells and up-regulated in sporulating cells during development [92].

Of the 1608 putative MrpC binding sites identified by our ChIP-seq analysis, 1059 are located in promoter regions, which we defined as the regions between -400 and +100 relative to predicted TSCs, and in 334 cases there are presumably divergent promoters, so the 1059 putative MrpC binding sites are located in the promoter regions of 1393 genes (Figure 7). Of course, there is uncertainty in predicting TSCs and in most cases the transcription start site is unknown. Despite these uncertainties, 133 of the putative MrpC binding sites are located in the promoter region of a gene up-regulated during development (Additional file 7, column C), based on the DNA microarray experiments [58, 59]; a very significant over-representation (Table 5). In addition, 3 of the sites in promoter regions of known MrpC targets (*mrpC, fruA, fmgD*) (Table 2) and another 11 in the promoter regions of genes directly involved in fruiting body formation (*hpk37, pkn9, pkn1, prw, pkn6, actB, nfsA, nfsH, mrpA, espC, asgD*) (Additional file 7, column A) were not detected as up-regulated in the microarray experiments but have been shown to be up-regulated during development by other approaches (*lacZ* fusion, RT-PCR, and/or immunoblot analyses). These approaches and an additional microarray study have identified 41 other genes (discussed below) that are up-regulated during development and have a putative MrpC binding site nearby, so MrpC is implicated as a transcriptional activator of at least 174 genes during development (Figure 7). We anticipate this number will increase as more transcriptomic methods such as RNA-seq are used to study *M. xanthus* development and as more developmental genes are analyzed individually.

Among the 174 genes implicated to be activated by MrpC, 22 are located immediately upstream of genes known or predicted to code for PKs (13 HPKs, 2 hybrid HPK/RRs, 7 STPKs). In addition, one promoter is located immediately upstream of a gene predicted to code for an RR (MXAN_0524) that may be co-transcribed in an operon with MXAN_0525, which is predicted to code for an STPK (Figure 7). Presumably, some of these PKs sense extracellular signals from the environment and from co-developing *M. xanthus* cells, while others sense intracellular cues; however, none of the signals are known. Nevertheless, our finding that MrpC likely up-regulates transcription of a large number of PKs suggests that an important function of MrpC is to heighten the sensory awareness of cells during the developmental process. In striking contrast, our results do not implicate MrpC in down-regulation of any PK (Figure 7).

Among the 22 (or 23 counting MXAN_0525) PKs whose transcription is likely up-regulated by MrpC, 13 have been characterized to some extent (Figure 7). Mutations in *pkn1* [71], *pkn6* [72], *pkn9* [70], *sdeK* [61, 93], *pskA12* [12], *hpk8* and *hpk37* [59], impair development, but the phosphorylation target proteins of these PKs have not been reported, although SdeK is known to be required for aggregation and activation of C-signal-dependent genes [61, 93] (Additional file 11A). AsgD has been implicated in A-signal production early in development [69] (Additional file 11A), but its target is also unknown. The target of Pkn8 is Pkn14, and Pkn14 phosphorylates MrpC, negatively regulating its accumulation during growth and development [18], and greatly reducing its binding to DNA [44]. Therefore, up-regulation of *pkn8* by MrpC activates a potential negative feedback loop to diminish MrpC activity if the (unknown) signal to which Pkn8 responds appears (Additional file 11B). Likewise, up-regulation of *espA*, *espC*, and *pktA5* by MrpC activates a potential negative feedback loop (Additional file 11B). In this case, EspA and EspC are hybrid HPKs with a receiver domain, and EspA is known to phosphorylate both its own receiver domain and that of EspC [49]. PktA5 appears to act together with PktA8 and EspB, and the complex may in turn interact with EspA [62]. The output of this complicated signaling system is to negatively regulate MrpC by increasing its proteolytic turnover [17, 49]. In contrast, up-regulation of the *mrpAB* operon by MrpC activates a potential positive feedback loop, since phosphorylated MrpB appears to activate transcription of *mrpC* [28] (Additional file 11B). However, it has been proposed that MrpB is phosphorylated by an unknown HPK and that MrpA, rather than acting as an HPK, dephosphorylates MrpB. Hence, up-regulation of *mrpA* by MrpC may also activate a potential negative feedback loop (Additional file 11B). Multiple mechanisms to negatively regulate MrpC activity appear to be important for preventing premature sporulation outside of fruiting bodies [17, 94]. Some of these mechanisms might also permit developing cells to halt commitment to sporulation if nutrients reappear [95].

Among the 174 genes implicated to be activated by MrpC, 14 are located immediately upstream of genes known or predicted to code for transcription factors (5 RRs including FruA, 5 DNA-binding proteins including MrpC, 3 σ factors, 1 EBP). In addition to FruA and MrpC, 4 others have been characterized to some extent (Figure 7). SigC negatively regulates development [63], but how it does so is unknown. ActB is part of the EBP cascade module [16] that plays several crucial roles in development (Additional file 11A), but whether the putative MrpC binding site located at -353 relative to the predicted *actB* TSC (Additional file 3) contributes to the observed up-regulation of the *actABCD* operon [74] remains to be seen. AsgB (MXAN_2913) is needed for A-signal production during development [96, 97] (Additional file 11A) and AsgB appears to be essential for growth [98]. Expression of an *asgB-lacZ* fusion increased about twofold by 13.75 h into development [98], possibly due to MrpC binding to a site located at -288 relative to the predicted TSC of *asgB* (+279 relative to that of MXAN_2912 in Additional file 3). Xre228 (MXAN_0228 in Additional file 7, column C) regulates genes involved in phase variation [99]. A putative MrpC binding site is located at -70 relative to the predicted MXAN_0228 TSC (Additional file 3). However, the significance of the implied up-regulation by MrpC is unclear since disruption of MXAN_0228 had little effect on development [99].

Examination of a second microarray study that identified 49 genes that are up-regulated at least 2.5-fold by 12 h into development [100] revealed 5 more genes whose promoters might be activated by MrpC. Four of these are predicted to code for transcription factors (MXAN_1970, 2902, 5041, 5899) and the fifth codes for C-signal (*csgA*, MXAN_1294). Therefore, MrpC is implicated to up-regulate 18 promoters that are located immediately upstream of genes known or predicted to code for transcription factors (5 RRs, 7 DNA-binding proteins, 3 σ factors, 3 EBPs) (Figure 7). Among the 4 additional genes predicted to code for transcription factors, MXAN_2902 (aka Mx_3320) was on our list of potentially interesting genes (Additional file 6) because a null mutant made fruiting bodies of abnormal shape under certain conditions in a previous study [100]. MXAN_2902 appears to code for an EBP involved in nitrogen sensing during development. MXAN_5041 (aka Mx_3098) is also predicted to code for an EBP but a null mutant showed no developmental defect [100]. Characterization of MXAN_1970 and 5899 has not been reported.

### MrpC is implicated as a direct activator or repressor of genes involved in signal production, spore formation, protein fate, and motility during development

Three of the up-regulated genes mentioned above are involved in extracellular signal production. AsgD and AsgB are a putative PK and transcription factor, respectively, involved in production of extracellular A-signal early in development [69, 96–98] (Additional file 11A). CsgA appears to be N-terminally truncated at the cell surface to produce C-signal [35–38] (Additional file 11A). The putative MrpC binding site located at +327 relative to the predicted TSC of MXAN_1293 (Additional file 3) is at -407 relative to that of the divergent *csgA* gene. While this site is slightly beyond the -400 cutoff we used to define likely regulatory sites, full expression of a *csgA-lacZ* fusion during development required DNA extending to -930 [101]. Therefore, MrpC bound to the site in MXAN_1293 might boost *csgA* expression.

Interestingly, other genes involved in C-signal production might also be up-regulated by MrpC. MXAN_0207 codes for PopD, an inhibitor of the protease (PopC) that cleaves CsgA at the cell surface [32] (Additional file 11A). The *popCD* operon was found to be up-regulated in microarray experiments [58, 59]. There is a putative MrpC binding site located at -227 relative to the predicted *popD* TSC (Additional file 3), so MXAN_0207 is among the up-regulated and potentially interesting genes with a putative MrpC binding located between -400 and +100 relative to its predicted TSC (Additional file 7, columns C and D). This site is within the *popC* coding region at +1210 relative to its predicted TSC. Intriguingly, there are three other putative MrpC binding sites within *popC* at +162, +450, and +1031 relative to its predicted TSC (Additional file 3; the +1031 site is at -406 relative to the predicted *popD* TSC). Whether these sites account for the observed up-regulation of *popCD* during development remains to be tested. Another gene involved in C-signal production and therefore on our list of potentially interesting genes (Additional file 6) is MXAN_4333, which codes for FtsH^D^, a protease important for degradation of PopD [32] (Additional file 11A). This proteolysis is regulated by ppGpp signaling and it releases PopC for secretion to the cell surface where it cleaves CsgA to produce C-signal [32]. Expression of *ftsH*^*D*^ increases early in development [32]. Interestingly, MXAN_4333 has a putative MrpC binding site located at -200 relative to its predicted TCS (Additional file 3). Therefore, MrpC might boost C-signal production by activating transcription of both *csgA* and components of the proteolytic cascade that acts on CsgA to produce C-signal.

The *bsgA* gene (MXAN_3993) codes for a protease that is believed to be involved in production of B-signal during development [50, 51] and was therefore on our list of potentially interesting genes (Additional file 6). Our ChIP-seq analysis identified a putative MrpC binding site located at -47 relative to the predicted *bsgA* TSC (Additional file 3). The start site of transcription has not been mapped, but expression of a *lacZ* fusion to *bsgA* (aka *lonD*) increased gradually during development [51]. The increase was less than twofold, so it is not surprising that it was not detected in microarray experiments. Nevertheless, the results suggest that MrpC might weakly activate transcription of *bsgA*, which in addition to its role in B-signal production also is necessary for production of MrpC2 [44] (Additional file 11B).

The *esgAB* locus (MXAN_4564 and 4565) codes for the subunits of a branched-chain keto acid dehydrogenase implicated in production of E-signal during development [102]. The two genes likely form an operon and were on our list of potentially interesting genes (Additional file 6). The upstream MXAN_4564 gene was found to be down-regulated during development in microarray experiments [58, 59]. Relative to the predicted TSC of this gene, we found a putative MrpC binding site located at +24 (Additional file 3), so MXAN_4564 is one of the down-regulated and potentially interesting genes in Additional file 7 (columns B and D). We infer that MrpC might repress transcription of the *esgAB* operon, inhibiting E-signal production (Figure 7). The Esg enzyme appears to be involved in synthesis of branched-chain fatty acids [102] that contribute to formation of unusual iso-branched ether lipids [103], which function as energy storage compounds and signals during development [104, 105]. Since MXAN_1676, 1675 (*plsB2*), the likely co-transcribed 1674, and/or the separately transcribed 5208 (*socA1*) had been proposed to be involved in ether lipid synthesis [106, 107], these genes were on our list of potentially interesting genes (Additional file 6). MXAN_1676 and *socA1* have putative MrpC binding sites located at +8 and -61 relative to their predicted TSCs, respectively (Additional file 3), suggesting these genes might be regulated by MrpC. The *socA* locus was initially identified by mutations that partially suppress the developmental defect of a *csgA* mutant [108]. The mutations cause overexpression of *socA1* [92], but how this rescues defective C-signaling is unclear [109]. Expression of *socA1* is down-regulated in rod-shaped cells and up-regulated in sporulating cells during development [92]. Recently, a cluster of five genes (MXAN_1531-1527) that likely form an operon has been shown to be responsible for ether lipid synthesis [110]. Interestingly, a putative MrpC binding site is located at +10 relative to the predicted TSC of MXAN_1530 (Additional file 3), and this gene was found to be down-regulated during development in microarray experiments [58, 59] (Additional file 7, column B). Therefore, MrpC might repress transcription of the MXAN_1531-1527 operon (Figure 7). All together, our results suggest that MrpC down-regulates E-signal production during development.

Several genes that regulate ppGpp-signaling are candidates for direct regulation by MrpC. In addition to its central role in C-signaling, CsgA induces the stringent response at the onset of development [111]. Hence, up-regulation of *csgA* by MrpC as described above may increase ppGpp signaling (Additional file 11A). In contrast, SocE inhibits the stringent response [111] (Additional file 11A). Early in development, the stringent response negatively regulates *socE* and positively regulates *csgA* [31]. The *socE* gene (MXAN_0731) was on our list of potentially interesting genes (Additional file 6) and a putative MrpC binding site was found at -231 relative to the predicted TSC (Additional file 3). Whether MrpC reinforces down-regulation of *socE* from this site remains to be explored. Like SocE, Nsd inhibits ppGpp accumulation when nutrients are available (Additional file 11A); however, the *nsd* gene is up-regulated at the onset of development [112]. A putative MrpC binding site was found at +40 relative to the predicted TSC of *nsd* (MXAN_7402) (Additional files 3 and 6).

Our results implicate MrpC in the up-regulation of a major spore protein, Prw (aka Protein W) [65], and in the regulation of several proteins important for spore formation (Figure 7 and Additional file 11A). A putative MrpC binding site was found at -335 relative to the predicted *prw* (MXAN_2491) TSC (+200 relative to that of MXAN_2490; Additional file 3). Nfs proteins are involved in deposition of the polysaccharide spore coat [75]. The locations of two putative MrpC binding sites upstream of the *nfs* operon (MXAN_3371-3378) and one within it, upstream of the last gene, *nfsH*, were noted in the results. Here, we note that a sizable gap between *nfsG* and *nfsH* could accommodate a suboperonic promoter regulated by MrpC from the site upstream of *nfsH*. Likewise, a suboperonic promoter could be regulated by MrpC from a putative binding site located at +45 relative to the predicted *exoE* TSC (Additional file 3) in the nine-gene *exo* operon (MXAN_3225-3233) (Additional file 6), whose products are also involved in deposition of the polysaccharide spore coat [75]. The *exo* operon is up-regulated during development [113] by FruA (Additional file 11B), which binds upstream of the operon promoter [55]. Sap proteins are small acid-soluble proteins important for resistance of spores to ultraviolet light [114]. The *sapA* (MXAN_7407) and *sapB* (MXAN_3885) genes were reported to be down- and up-regulated, respectively, during development in microarray experiments [58, 59] (Additional file 7). Putative MrpC binding sites were found at -39 and -365 relative to the predicted TSC of *sapA* and *sapB*, respectively (Additional file 3).

Our results implicate MrpC in the down- or up-regulation of genes involved in protein fate and motility during development, but most of these genes have not been studied much (Figure 7). Of the 13 genes (listed in results) predicted to control the fate of other proteins, shown to be down-regulated during development in microarray experiments [58, 59] (Additional file 7, column B), and having a putative MrpC binding site located between -400 and +100 relative to the predicted TCS, none to our knowledge have been knocked out to test for a developmental defect. On the other hand, most of the genes involved in motility were identified as such by mutational analysis, but only a few are known to be down-regulated (*gltB, sgmH, efp, epsL, agmO*) or up-regulated (*cglD, aglCR, sgmK*) during development, based on microarray experiments [58, 59] (Additional file 7; see Additional file 6 for gene numbers). Two others have been studied in some detail. PilA codes for pilin, the structural component of type IV pili, which are needed for S motility [115]. The *pilA* gene (MXAN_5783) is first up-regulated and then down-regulated during development [90]. Two putative MrpC binding sites, located at -173 and -743 relative to the predicted *pilA* TSC (Additional file 3), might participate in its developmental regulation. The *che4* operon (MXAN_2681-2686) is up-regulated during development and its products form a chemosensory system that regulates S motility [116]. A putative MrpC binding site located at -140 relative to the predicted *cheW4a* TSC (-105 relative to that of MXAN_2680; Additional file 3) might mediate the observed up-regulation. In addition to these 10 genes or operons, another 24 involved in motility have a putative MrpC binding site located between -400 and +100 relative to their predicted TCS (see Additional file 7, column D; Additional file 6, description and process) but either developmental regulation was missed in the microarray experiments or MrpC does not regulate these genes. Noteworthy among these genes or operons are the *che6* (MXAN_6947-6954) and *che7* (MXAN_6965-6958) operons, which code for chemosensory systems in which mutations cause defects in S motility and development [117]. Putative MrpC binding sites were found at -55 and -80 relative to the predicted TSCs of MXAN_6947 and 6965, respectively, the first gene of each operon (Additional files 3 and 6). Although the *che5* cluster of genes (MXAN_6033-6027) has not been analyzed thoroughly, a mutation in *cheA5* (MXAN_6029) caused premature development [117]. A putative MrpC binding site is located at -33 relative to the predicted TSC of the first gene (MXAN_6033) of this putative operon (Additional files 3 and 6). Also worth noting are two putative MrpC binding sites in the vicinity of *epsL* (MXAN_7437, aka *czc3A*; Additional file 6), a gene noted above as down-regulated during development in microarray experiments [58, 59] (Additional file 7, column B). A putative MrpC binding site located at +65 relative to the predicted *epsL* TCS (Additional file 3) might account for the down-regulation. The second putative MrpC binding site is located at -95 relative to the predicted TSC of *epsK* (MXAN_7438, aka *czc3B*; Additional file 6) (-35 relative to the predicted TSC of MXAN_7439; Additional file 3). The *epsK* gene is likely co-transcribed with *epsL* (Additional file 6)*.* Both genes are implicated in S motility [118] and in efflux of heavy metal ions [91]. Expression of *epsL* (measured from a *czc3B-lacZ* fusion) increased early in development and then decreased [91], so MrpC might be involved in this regulation. Alternatively or in addition, MrpC binding to the same site might regulate the divergent promoter of MXAN_7439 (*epsJ*), which is likely co-transcribed with *nla24* (MXAN_7440, aka *epsI*; Additional file 6). Both of these genes are also implicated in S motility [119], with Nla24 being an EBP [23, 120].

### Binding of MrpC and FruA to DNA

Bioinformatic analysis of the 500 top-ranked putative MrpC binding sites revealed a motif that strongly resembles one of two consensus sequences for MrpC binding proposed previously [44]. The motif is the imperfect palindrome TGTYN_8_RAC (Figure 3B). We tried to use this motif to identify putative MrpC binding sites genome-wide, but parameters that retained known sites yielded large numbers of predicted sites. Our bioinformatic analysis of the top 500 putative MrpC binding sites did not identify Motif 1 (Figure 3A), which was proposed previously based on six sequences in the *mrpC* promoter region to which MrpC bound [44]. Perhaps Motif 1 is too short and too degenerate to be detected by our analysis. We thought our analysis might uncover a motif related to FruA binding, since FruA binds cooperatively with MrpC at several sites [45–48, 53]. A consensus sequence for binding of the FruA DNA-binding domain, GGGYRN_4-6_YGGG has been proposed [121], but MEME discovered no motif resembling this sequence in the vicinity of the top 500 putative MrpC binding sites. It is possible that cooperative interactions with MrpC allow FruA to bind to sequences dissimilar to the proposed consensus.

Our finding that His_10_-MrpC and His_10_-MrpC2 bind to a region upstream of the *fruA* promoter with similar affinity is surprising (Additional file 8). A previous comparison of the two proteins suggested that MrpC2 has 8-fold and 4-fold higher affinity for the *fruA* and *mrpC* promoter regions, respectively [44]. Although we do not understand this discrepancy, our finding does not preclude an important role for the conversion of MrpC to MrpC2 during development. MrpC2 cannot be phosphorylated by Pkn14, and MrpC-P has been reported to be unstable [18] and to bind DNA poorly [44]. However, it was found recently that under certain conditions of development, MrpC2 accumulation is diminished at least temporarily with no discernible impact on development [95]. The precise roles of MrpC and MrpC2 during development remain to be defined.

MrpC2 bound to DNA containing a putative MrpC binding site in every case we tested. These cases included top-ranked ChIP-seq peaks as well as low-ranked peaks (Additional file 9). DNA corresponding to all the top-ranked peaks, as well as several lower-ranked peaks, produced multiple shifted complexes in EMSAs, indicative of multiple MrpC2 binding sites (Figure 4 and Additional file 9). Presumably, as noted above, both the number and affinity of binding sites contribute to detectability and hence rank in the ChIP-seq analysis. Binding of other proteins is also expected to affect detectability, negatively if the two proteins compete for binding to overlapping sites, or positively if the two proteins bind cooperatively. FruA binds cooperatively with MrpC2 at several sites [45–48, 53]. In one case, the two proteins appear to compete for binding to overlapping sites [45].

MrpC2 bound to DNA sequences matching the motif identified by our bioinformatic analysis (Figure 3B) in all 11 cases tested (Figure 5). In one case, two matches to the motif were found near one of the putative MrpC binding sites (MXAN_0524, Additional file 9). Both sequences matching the motif were bound by MrpC2, but the sequence in between and containing only half of each match to the motif was not bound (Figure 5, lanes 1-6). We conclude that the motif is highly predictive of MrpC2 binding. On the other hand, MrpC2 also binds to DNA sequences that do not match the motif very well. Several 200-bp DNA fragments containing a putative MrpC binding site but with no strong match to the motif nevertheless were bound by one or more MrpC2, and several fragments with one match to the motif were bound by two or more MrpC2 (Additional file 9). In some cases, cooperative interactions between MrpC2 dimers might facilitate binding, although this remains to be tested.

We found evidence for cooperative binding of MrpC2 and FruA in 13 of 15 cases tested (Figure 6; Additional file 10; summarized in Additional file 9). Together with previous work on five promoter regions that are up-regulated during development sites [45–48, 53], the data strongly suggest that cooperative binding of the two transcription factors is a pervasive regulatory mechanism during *M. xanthus* development. This cooperativity was proposed to allow cells to monitor both aggregation and nutritional status before committing to sporulation [46]. FruA activity appears to increase in response to C-signaling as cells aggregate and become aligned in nascent fruiting bodies [39–42]. Recently, MrpC was shown to be sensitive to nutrient-regulated proteolysis before and during the critical period of commitment to sporulation [95]. Only if cells are starving and close-packed in mounds are both MrpC and FruA active enough to permit transcription of genes that commit cells to spore formation, resulting in mature fruiting bodies.

Several patterns of cooperative MrpC2 and FruA binding were observed. In the first pattern, there was little binding of FruA alone, but in combination with MrpC2 much more of a slower-migrating complex was formed than with MrpC2 alone, although MrpC2 alone bound strongly (Figure 6, lanes 2-4; Additional file 10, top row). In these cases, bound MrpC2 appeared to enhance the binding of FruA. Except for the site within MXAN_4360, these sites are upstream of predicted TSCs of genes up-regulated during development and/or known to be involved in development (Additional file 9). In the second pattern, each protein bound strongly alone, and FruA appeared to be binding to multiple sites (Figure 6, lanes 8-10; Additional file 10, lanes 18-20 and 26-28). FruA may be binding cooperatively to these sites, although this remains to be tested. The strong binding by both proteins made it difficult to determine whether cooperative binding was occurring at our standard protein concentrations. By testing lower concentrations of the two proteins, there appeared to be more than simply additive binding (Figure 6, lanes 12-14; Additional file 10, lanes 22-24). In the case of the *bsgA* upstream region, we expected to see a novel, slow-migrating complex in lane 14 of Figure 6, due to binding of MrpC2 and FruA. Instead, more of a complex that co-migrated with the complex produced by MrpC2 alone was observed. We speculate that the complex with both proteins bound is unstable or migrates aberrantly. In any case, regions exhibiting the second pattern of binding may rely on cooperative interactions between MrpC2 and FruA early in development when their concentrations are low, but not later when their concentrations are high. Of the three regions exhibiting this pattern, *bsgA* is weakly up-regulated during development and codes for a protease involved in B-signaling [50, 51], MXAN_6247 is down-regulated during development [58, 59], and *cheW6a* is the first gene of an operon that codes for a chemosensory system involved in S motility and development [117]. In the third pattern, FruA alone failed to produce a distinct complex, but there was evidence of cooperative binding in combination with MrpC2 (Figure 6, lanes 16-20; Additional file 10, third row). This pattern is similar to the first pattern, in which bound MrpC2 appears to enhance the binding of FruA. Notable among the genes exhibiting the third pattern are *mrpC* itself and *pkn8*. Hence, our results for the first time implicate FruA in feedback loops that regulate MrpC.

## Conclusion

We conclude that MrpC binds to the promoter regions of hundreds of developmentally-regulated genes of *M. xanthus* at 18 h poststarvation. In many cases, MrpC likely binds cooperatively with FruA, subjecting transcription of target genes to both nutritional and morphological cues. The implied targets of activation by MrpC alone or in combination with FruA include 23 PKs, 18 transcription factors, 8 genes involved in production of A-, C-, and ppGpp-signals, 15 genes involved in spore formation, and 12 genes involved in motility (Figure 7). Other genes involved in signal production (e.g., E-signal) or motility, as well as 13 that control the fate of other proteins, may be repressed by MrpC. The profound effects of MrpC on developmental gene expression, including activation of *fruA* transcription and combinatorial control with FruA of downstream targets and feedback loops, predict that many indirect effects will be observed in comparative transcriptomic analysis of wild type with *mrpC* and *fruA* mutants. Care in interpreting such data will be necessary. This type of further work would be most useful if done in a way that identified transcript 5’ ends (potential transcription start sites) genome-wide, which is possible using RNA-seq approaches [122]. Performing ChIP-seq analysis for both MrpC and FruA at additional times poststarvation is another clear direction for future experiments. This first report of genome-wide binding analysis for a transcription factor in *M. xanthus* yielded a plethora of predictions about the role of MrpC in regulating developmental genes, which will need to be tested.

## Methods

### Bacterial strain, growth, and development

*M. xanthus* wild-type strain DK1622 [123] was grown at 32°C in CTT (1% Casitone, 10 mM Tris-HCl [pH 8.0], 1 mM KH_2_PO_4_-K_2_HPO_4_, 8 mM MgSO_4_ [final pH, 7.6]) broth [124] or on CTT agar (1.5%) plates. Fruiting body development was performed on TPM (10 mM Tris-HCl [pH 8.0], 1 mM KH_2_PO_4_-K_2_HPO_4_, 8 mM MgSO_4_ [final pH, 7.6]) agar (1.5%) plates, as described previously [125]. Under these conditions, mounds have formed by 18 h [126], but spores have not yet appeared (L. Kroos, unpublished observations).

### ChIP

After 18 h of fruiting body development, cells were collected and subjected to ChIP, as described previously [46, 127], with anti-MrpC antibodies (500 ng) [44] or control IgG (500 ng) (Santa Cruz Biotechnology). Briefly, cells were treated with formaldehyde to cross-link proteins to DNA, the cell suspension was sonicated, the lysate was microcentrifuged, the supernatant was pretreated with protein A-Sepharose beads to minimize subsequent nonspecific binding, the supernatant was incubated with antibodies and then with protein A-Sepharose beads for immunoprecipitation, the beads were collected by microcentrifugation and washed, the cross-links were reversed, the proteins were digested, and the DNA was purified [127]. The resulting DNA was analyzed by PCR with primers from -101 to +25 of the *fmgA* (previously Ω4400) promoter region, as described previously [127], to confirm enrichment in the sample with anti-MrpC IgG compared with control IgG.

### DNA sequencing

Samples of DNA (~10 ng) resulting from ChIP were processed using a ChIP-seq sample preparation kit according to the manufacturer’s instructions (Illumina). Briefly, the DNA ends were repaired to produce blunt-ended fragments, the DNA was treated with Klenow fragment to generate 3’-dA overhangs, and oligonucleotide adapters were ligated onto the DNA ends. Each sample was size-selected by excising and extracting fragments of approximately 300 bp after electrophoresis on a 2% agarose gel, the DNA fragments were enriched by PCR, and the library was validated on an Agilent Technologies 2100 Bioanalyzer. Sequencing was performed at the Michigan State University Research Support Technology Facility using a kit designed to produce reads of 36 nucleotides (Illumina) and a Solexa instrument. The DNA sequence reads have been deposited at the NCBI Sequence Read Archive under accession number SRP049504.

### Detection of ChIP-seq peaks

DNA sequence reads were aligned to the *M. xanthus* genome [10] using the short-read alignment software package Bowtie [128]. Genomic regions with significant enrichment of aligned reads in the anti-MrpC ChIP sample compared with the control IgG ChIP sample in each of two experiments were detected using the kernel density estimator-based analysis package QuEST [52]. A partition of the reads from the IgG ChIP sample was set aside in each experiment and treated as if it was a ChIP-enriched sample to allow estimation of the false-positive rate of the peak detection process.

### Analysis of ChIP-seq peak locations

Custom, in-house Python scripts were written to determine proximity of ChIP-seq peaks to genomic features of interest, such as the predicted TSC of the nearest gene, as well as to generate Monte-Carlo simulations of the data for comparison with randomly placed peaks in the genome. Functional annotation and categorization of *M. xanthus* genes was assigned by the J. Craig Venter Institute and amended as described previously [57]. The scripts and associated input files (e.g., genome and annotations) that they rely on are available at https://github.com/blobbybirdman/MrpC_MXanthus. Lists of genes were compared using a custom Java application written in-house.

### Identification of a DNA sequence motif in putative MrpC binding sites

To assess the presence of conserved sequence motifs associated with ChIP-seq peaks enriched in the anti-MrpC sample, flanking sequence (50 bp on each side) was extracted for each peak in order to allow for positional uncertainty of the peak and to allow the possibility of detecting motifs of other factors that might interact with MrpC. The full dataset was ranked by peak enrichment and was then partitioned into sets of 50 sequences to allow the discrimination of motifs associated with strong versus weak MrpC binding sites if present. The top 500 ranked peaks and the smaller dataset partitions were searched using MEME [76] to detect any overrepresented motifs. The motif of interest was compared with motifs in RegTransBase [78] using TOMTOM [76] to identify similarity to known transcription factor binding motifs.

### Protein purification

His_10_-MrpC and His_10_-MrpC2 were purified as described previously [18, 44] from *Escherichia coli* strain BL21(DE3) [129] transformed with pET-MrpC and pET-MrpC2, respectively. However, *E. coli* transformed with pET-MrpC grew slowly on Luria-Bertani (LB) [130] agar (1.5%) plates containing ampicillin (50 μg/ml) at 37°C, whereas *E. coli* transformed with pET-MrpC2 grew normally. By transferring fresh transformants into LB broth containing ampicillin (50 μg/ml) and growing at 37°C until the density reached 100 Klett units (about 5 × 10^8^ cells/ml), then inducing with isopropyl-1-thio-β-D-galactopyranoside (IPTG) (1 mM) for 1.5 h, expression of His_10_-MrpC could be observed, although not as high as that of His_10_-MrpC2, and each protein could be purified. FruA-His_6_ was purified as described previously [46] from *E. coli* BL21(DE3) transformed with pET11km/FruA-His_6_.

### EMSAs

DNA fragments were generated by PCR using primers listed in Additional file 12, purified using a PCR purification kit (Qiagen), and labeled with [γ-^32^P]ATP using T4 polynucleotide kinase (New England BioLabs). Alternatively, oligonucleotides were likewise ^32^P-labeled, mixed in pairs as listed in Additional file 12, and annealed by being allowed to cool to room temperature after incubation in a boiling water bath for 10 min. The ^32^P-labeled DNA probes were purified and used in EMSAs as described previously [127], except that binding reaction mixtures were incubated at 25°C for 15 min.

## Authors’ information

MR is currently a research associate at Benaroya Research Institute, 1201 Ninth Avenue, Seattle, WA 98101, USA. BS is currently a bioinformatics analyst at Theragen Bio Institute, South Korea. DK is currently a software developer in the Ford College Graduates Program, Ford Motor Company, Dearborn, MI 48126, USA.

## Electronic supplementary material

Additional file 1: **Signaling and gene regulatory network during** ***M. xanthus*** **development.** Diagram depicting dependence of three gene regulatory modules and fruiting body formation on four signals. (DOCX 19 KB)

Additional file 2: **The MrpC module.** Diagram emphasizing inputs into the MrpC module and feedback loops. (DOCX 40 KB)

Additional file 3: **ChIP-seq peaks.** The list of 1608 ChIP-seq peaks from two experiments showing overall rank (related to the number of sequence reads), peak coordinate (average genome coordinate of the two peak maxima), rank in each experiment, genome coordinate of each peak maximum, number of the gene whose predicted TSC is nearest the peak coordinate, gene functional annotation, gene start and stop coordinates, and position of the peak coordinate relative to the nearest predicted TSC. (XLSX 190 KB)

Additional file 4: **ChIP-seq peaks in the** ***mrpC*** **and** ***fruA*** **promoter regions.** Figure showing the relative peak height and position in the genome from two experiments. Also shown is the position of MrpC binding sites from *in vitro* studies of each promoter region. (DOCX 94 KB)

Additional file 5: **Distribution of putative MrpC binding sites near a predicted TSC.** Figure showing distances from putative MrpC binding sites to predicted TSCs as compared with sites placed randomly in the genome. (DOCX 65 KB)

Additional file 6: **Potentially interesting genes.** The list of genes involved in motility, signaling, or gene expression that may play a role in development, including gene number and name (if one has been assigned), description of the corresponding protein and the process in which it is involved (for genes with a putative MrpC binding site between -400 and +100 relative to their predicted TSC), and a reference(s). (DOCX 220 KB)

Additional file 7: **Developmental genes associated with putative MrpC binding sites.** The list of genes with a putative MrpC binding site between -400 and +100 relative to their predicted TSC, and known to be directly involved in fruiting body formation, down- or up-regulated during development, or designated as potentially interesting in Additional file 6. (XLS 50 KB)

Additional file 8: **Binding of MrpC2 and MrpC to the** ***fruA*** **promoter region.** Figure showing a comparison of purified proteins binding to a DNA fragment in EMSAs. (DOCX 47 KB)

Additional file 9: **Summary of His**_**10**_ **-MrpC2 and FruA-His**_**6**_ **binding to DNA fragments in EMSAs.** List of putative MrpC binding sites tested for binding of MrpC2 and FruA, including peak rank, gene number and name (if one has been assigned), position of the peak coordinate relative to the nearest predicted TSC, relevant characteristics, the apparent number of MrpC2 binding sites in EMSAs with fragments generated by PCR, the number of motifs bound in EMSAs with annealed oligonucleotides, whether FruA binding was observed in EMSAs with fragments generated by PCR, and whether there was evidence of cooperative binding of FruA and MrpC2. (DOCX 14 KB)

Additional file 10: **Patterns of MrpC2 and FruA binding to candidate genes from ChIP-seq.** Figure showing EMSAs of purified proteins binding to DNA fragments generated by PCR. (DOCX 429 KB)

Additional file 11: **Roles of genes implicated to be under direct control of MrpC in the signaling and gene regulatory network during** ***M. xanthus*** **development.** Diagrams depicting roles of putative MrpC-controlled genes in the MrpC module of the network and in the overall network. (DOCX 39 KB)

Additional file 12: **Primers for PCR and oligonucleotides for EMSAs.** The list of primers used to generate DNA fragments by PCR and the list of oligonucleotides that were annealed to produce probes for EMSAs. (DOCX 13 KB)
