# Supplementary material for: Transcription factor MrpC binds to promoter regions of hundreds of developmentally-regulated genes in Myxococcus xanthus
Source: BMC Genomics. 2014 Dec 16;15:1123. doi: 10.1186/1471-2164-15-1123 (PMC4320627; doi:10.1186/1471-2164-15-1123)
Supplement: Supplementary file 1 — Additional file 1: Signaling and gene regulatory network during M. xanthus development. Diagram depicting dependence of three gene regulatory modules and fruiting body formation on four signals. (DOCX 19 KB) [file 12864_2014_6823_MOESM1_ESM.docx]

**Additional file 1 Signaling and gene regulatory network during *M. xanthus* development.** Starvation initiates intracellular ppGpp signaling, which leads to extracellular A- and C-signaling. Starvation also initiates the EBP cascade module and the Mrp module. The EBP cascade module promotes the Mrp module and all three signals. Output of the Mrp module is MrpC and MrpC2, which activate transcription of *fruA* and together with activated FruA (designated FruA*), activate transcription of genes important for fruiting body formation. See the text for references and see [1] for a more detailed description of the regulatory network.

**Reference**

1. Rajagopalan R, Sarwar Z, Garza AG, Kroos L: **Developmental gene regulation**. In: *Myxobacteria: genomics, cellular and molecular biology.* Edited by Yang Z, Higgs P. Norfolk, UK: Caister Academic Press; 2014: 105-126.
