# Supplementary material for: Transcription factor MrpC binds to promoter regions of hundreds of developmentally-regulated genes in Myxococcus xanthus
Source: BMC Genomics. 2014 Dec 16;15:1123. doi: 10.1186/1471-2164-15-1123 (PMC4320627; doi:10.1186/1471-2164-15-1123)
Supplement: Supplementary file 2 — Additional file 2: The MrpC module. Diagram emphasizing inputs into the MrpC module and feedback loops. (DOCX 40 KB) [file 12864_2014_6823_MOESM2_ESM.docx]

**Additional file 2 The MrpC module.** Starvation triggers the EBP cascade [1] and an increase in intracellular (p)ppGpp [2, 3], causing induction of the *mrpAB* operon [4]. MrpA is a putative histidine protein kinase/phosphatase thought to influence phosphorylation of MrpB, a putative EBP hypothesized to positively autoregulated the *mrpAB* operon and activate *mrpC* transcription [5]. Other mechanisms linking MrpC activity to starvation include the Esp signaling system [6, 7] and two STPKs (Pkn8 and Pkn14) that govern phosphorylation of MrpC [8]. MrpC is converted to an N-terminally-truncated form, MrpC2, in a process that requires BsgA and might be inhibited by phosphorylation of MrpC [9, 10]. It has been reported that MrpC positively autoregulates and positively regulates *mrpAB* [5]. Positive feedback loops are green. For simplicity, only MrpC is depicted to positively autoregulate and positively regulate *mrpAB*, but MrpC2 likely does as well. This figure was adapted from [11] with permission.

**References**

1. Giglio KM, Caberoy N, Suen G, Kaiser D, Garza AG: **A cascade of coregulating enhancer binding proteins initiates and propagates a multicellular developmental program**. *Proc Natl Acad Sci USA* 2011, **108**:E431-E439.

2. Manoil C, Kaiser D: **Accumulation of guanosine tetraphosphate and guanosine pentaphosphate in *Myxococcus xanthus* during starvation and myxospore formation**. *J Bacteriol* 1980, **141**:297-304.

3. Manoil C, Kaiser D: **Guanosine pentaphosphate and guanosine tetraphosphate accumulation and induction of *Myxococcus xanthus* fruiting body development**. *J Bacteriol* 1980, **141**(1):305-315.

4. Sun H, Shi W: **Analyses of *mrp* genes during *Myxococcus xanthus* development**. *J Bacteriol* 2001, **183**(23):6733-6739.

5. Sun H, Shi W: **Genetic studies of *mrp*, a locus essential for cellular aggregation and sporulation of *Myxococcus xanthus***. *J Bacteriol* 2001, **183**(16):4786-4795.

6. Higgs PI, Jagadeesan S, Mann P, Zusman DR: **EspA, an orphan hybrid histidine protein kinase, regulates the timing of expression of key developmental proteins of *Myxococcus xanthus***. *J Bacteriol* 2008, **190**:4416-4426.

7. Schramm A, Lee B, Higgs PI: **Intra- and inter-protein phosphorylation between two hybrid histidine kinases controls *Myxococcus xanthus* developmental progression**. *J Biol Chem* 2012, **287**:25060–25072.

8. Nariya H, Inouye S: **Identification of a protein Ser/Thr kinase cascade that regulates essential transcriptional activators in *Myxococcus xanthus* development**. *Mol Microbiol* 2005, **58**(2):367-379.

9. Nariya H, Inouye S: **A protein Ser/Thr kinase cascade negatively regulates the DNA-binding activity of MrpC, a smaller form of which may be necessary for the *Myxococcus xanthus* development**. *Mol Microbiol* 2006, **60**(5):1205-1217.

10. Ueki T, Inouye S: **Identification of an activator protein required for the induction of *fruA*, a gene essential for fruiting body development in *Myxococcus xanthus***. *Proc Natl Acad Sci USA* 2003, **100**(15):8782-8787.

11. Rajagopalan R, Sarwar Z, Garza AG, Kroos L: **Developmental gene regulation**. In: *Myxobacteria: genomics, cellular and molecular biology.* Edited by Yang Z, Higgs P. Norfolk, UK: Caister Academic Press; 2014: 105-126.
