# Supplementary material for: Transcription factor MrpC binds to promoter regions of hundreds of developmentally-regulated genes in Myxococcus xanthus
Source: BMC Genomics. 2014 Dec 16;15:1123. doi: 10.1186/1471-2164-15-1123 (PMC4320627; doi:10.1186/1471-2164-15-1123)
Supplement: Supplementary file 4 — Additional file 4: ChIP-seq peaks in the mrpC and fruA promoter regions. Figure showing the relative peak height and position in the genome from two experiments. Also shown is the position of MrpC binding sites from in vitro studies of each promoter region. (DOCX 94 KB) [file 12864_2014_6823_MOESM4_ESM.docx]

**Additional file 4 ChIP-seq peaks in the *mrpC* and *fruA* promoter regions.** (A) ChIP-seq peak in the *mrpC* promoter region from two experiments. Below the graphs is a map drawn to scale with numbering relative to the translation start codon (+1) and a bent arrow at the position of the transcriptional start site [1]. A dashed line indicates the position of at least 6 MrpC binding sites [2]. (B) ChIP-seq peak in the *fruA* promoter region from two experiments. Below the graphs is a map drawn to scale using the same conventions as in panel A and indicating the positions of the transcriptional start site [3] and 2 MrpC binding sites [4].

**References**

1. Nariya H, Inouye S: **Identification of a protein Ser/Thr kinase cascade that regulates essential transcriptional activators in *Myxococcus xanthus* development**. *Mol Microbiol* 2005, **58**(2):367-379.

2. Nariya H, Inouye S: **A protein Ser/Thr kinase cascade negatively regulates the DNA-binding activity of MrpC, a smaller form of which may be necessary for the *Myxococcus xanthus* development**. *Mol Microbiol* 2006, **60**(5):1205-1217.

3. Ogawa M, Fujitani S, Mao X, Inouye S, Komano T: **FruA, a putative transcription factor essential for the development of *Myxococcus xanthus***. *Mol Microbiol* 1996, **22**(4):757-767.

4. Ueki T, Inouye S: **Identification of an activator protein required for the induction of *fruA*, a gene essential for fruiting body development in *Myxococcus xanthus***. *Proc Natl Acad Sci USA* 2003, **100**(15):8782-8787.
