# Supplementary material for: Transcription factor MrpC binds to promoter regions of hundreds of developmentally-regulated genes in Myxococcus xanthus
Source: BMC Genomics. 2014 Dec 16;15:1123. doi: 10.1186/1471-2164-15-1123 (PMC4320627; doi:10.1186/1471-2164-15-1123)
Supplement: Supplementary file 5 — Additional file 5: Distribution of putative MrpC binding sites near a predicted TSC. Figure showing distances from putative MrpC binding sites to predicted TSCs as compared with sites placed randomly in the genome. (DOCX 65 KB) [file 12864_2014_6823_MOESM5_ESM.docx]

**Additional file 5 Distribution of putative MrpC binding sites near a predicted translation start codon (TSC).** (A) Distances from putative MrpC binding sites to predicted TSCs. The 1608 sites were filtered to include only those between -400 and +100 relative to a predicted TSC. The distribution of distances from sites to the nearest predicted TSC (or the two nearest predicted TSCs for sites associated with divergent genes) is plotted as a frequency histogram. (B) Distances from randomized peaks to predicted TSCs. Monte Carlo simulation was used to generate 1608 random sites within the genome. The sites were filtered as for panel A. Of the 541 remaining sites, 90 were associated with divergent genes. These numbers are smaller than for panel A since putative MrpC binding sites are preferentially located near predicted TSCs.
