# Supplementary material for: Transcription factor MrpC binds to promoter regions of hundreds of developmentally-regulated genes in Myxococcus xanthus
Source: BMC Genomics. 2014 Dec 16;15:1123. doi: 10.1186/1471-2164-15-1123 (PMC4320627; doi:10.1186/1471-2164-15-1123)
Supplement: Supplementary file 6 — Additional file 6: Potentially interesting genes. The list of genes involved in motility, signaling, or gene expression that may play a role in development, including gene number and name (if one has been assigned), description of the corresponding protein and the process in which it is involved (for genes with a putative MrpC binding site between -400 and +100 relative to their predicted TSC), and a reference(s). (DOCX 220 KB) [file 12864_2014_6823_MOESM6_ESM.docx]

**Additional file 6 Potentially interesting genes**

| MXAN^a^ | Gene | Description; process^b^ | Reference |
| --- | --- | --- | --- |
| 0001 | *dnaA* |  | [1] |
| 0172 |  |  | [2] |
| 0180 | *Mxa296* |  | [3] |
| 0203 |  |  | [4] |
| 0206 | *popC* |  | [5] |
| 0207 | *popD* | PopC inhibitor; C-signaling | [6] |
| 0228 | *xre228* | HTH-Xre-like DNA-binding protein; phase variation | [7] |
| 0267 | *pdeC* |  | [8] |
| 0358 | *sgmA, ileS* |  | [9] |
| 0440 | *sgmB* |  | [9] |
| 0552 | *pskA9* |  | [10] |
| 0581 | *dsg* |  | [11] |
| 0603 |  |  | [2] |
| 0635 | *agmE* |  | [9] |
| 0639 | *cyaB* | adenylyl cyclase; signaling | [12] |
| 0731 | *socE* | hypothetical protein; ppGpp-signaling | [13], L. Shimkets |
| 0785 | *sigF* | σ; S motility | [9] |
| 0907 |  |  | [2] |
| 0962 | *cglD* | lipoprotein; A motility | [14] |
| 0979 | *copC* |  | [15] |
| 1061 | *rpoN* | σ^54^; gene expression | [16] |
| 1076 | *selB* |  | [9] |
| 1078 | *spdR, nla19* |  | [17, 18] |
| 1079 | *prx* |  | [19] |
| 1106 | *sgmC* |  | [9] |
| 1130 | *frgA* |  | [20] |
| 1210 |  |  | [4] |
| 1245 | *sasR* |  | [21, 22] |
| 1272 | *pdeB* |  | [8] |
| 1286 | *abcA* |  | [23] |
| 1364 | *tag* |  | [24, 25] |
| 1450 | *oar* |  | [26] |
| 1510 |  |  | [4] |
| 1641 | *sgmD* |  | [9] |
| 1668 | *MkapC* |  | [27] |
| 1673 | *agmN* |  | [9] |
| 1674 |  |  | [28, 29] |
| 1675 | *plsB2* |  | [28, 29] |
| 1676 |  | 1-alkyldihyroxyacetone 3-phosphate synthase; lipid signaling | [28, 29] |
| 1795 | *sgmE* | putative liproprotein; S motility | [9] |
| 1915 | *Wza* |  | [9] |
| 1925 | *mglA* |  | [9, 30] |
| 1926 | *mglB* |  | [9, 30] |
| 1929 | *masK* | tyrosine protein kinase; S motility | [31] |
| 2030 |  | ECF σ; gene expression | [4] |
| 2044 | *pph1* |  | [32] |
| 2050 | *pglH* |  | [33] |
| 2128 | *sgmF* |  | [9] |
| 2203 | *sgmG* | hypothetical protein; hydrolase; S motility | [9] |
| 2252 | *ark* | arginine kinase; development | [34] |
| 2293 | *easB* |  | [9] |
| 2526 | *sgmH* | hypothetical protein; S motility | [9] |
| 2538 | *agmO, cglC, gltK* | lipoprotein; A motility | [9, 14, 35] |
| 2539 | *gltB* | hypothetical protein; A motility | [35] |
| 2540 | *gltA* |  | [35] |
| 2541 | *aglAR, gltC* |  | [35, 36] |
| 2542 | *agmP* |  | [9] |
| 2549 | *pkn5* | STPK; signaling | [37] |
| 2561 | *sgmI* |  | [9] |
| 2629 | *K9ap1* |  | [27] |
| 2670 | *asgA* |  | [38] |
| 2681 | *cheW4a* | coupling protein; signaling, S motility | [39] |
| 2682 | *cheR4* |  | [39] |
| 2683 | *mcp4* |  | [39] |
| 2684 | *cheY4* | RR; signaling, S motility | [39] |
| 2685 | *cheW4b* | coupling protein; signaling, S motility | [39] |
| 2686 | *cheA4* |  | [39] |
| 2703 | *pdeE* |  | [40] |
| 2723 | *cyaA* |  | [41] |
| 2902 | *Mx3320* | EBP; gene expression | [42] |
| 2913^c^ | *asgB* | DNA-binding protein; A-signaling | [43] |
| 2914 | *purH* |  | [9] |
| 2916 | *purD* |  | [9] |
| 2919 | *pglJ* |  | [33] |
| 2921 | *pglB, sgmJ* |  | [9, 33] |
| 2922 | *sgmK* | NDP-sugar epimerase; S motility | [9] |
| 2923 | *agmQ* |  | [9] |
| 2929 |  |  | [4] |
| 2957 | *sigD* |  | [44] |
| 2991 | *agmZ* |  | [9] |
| 3008 | *aglU* |  | [36] |
| 3055 | *agmB, hrpB* |  | [9] |
| 3060 | *cglB* |  | [9] |
| 3084 | *tgl* |  | [9] |
| 3122 | *bcsA* | putative monooxygenase; bypass of B- and C-signaling | [45] |
| 3190 | *sdmA* |  | [46] |
| 3225 | *exoA, fdgA* |  | [47, 48] |
| 3226 | *exoB* |  | [47] |
| 3227 | *exoC* |  | [47] |
| 3228 | *exoD, btkA* |  | [47, 49] |
| 3229 | *exoE* | sugar transferase; spore exopolysaccharide synthesis | [47] |
| 3230 | *exoF* |  | [47] |
| 3231 | *exoG* |  | [47] |
| 3232 | *exoH* |  | [47] |
| 3233 | *exoI* |  | [47] |
| 3352 | *agmF* |  | [9] |
| 3415 | *copA* |  | [15] |
| 3422 | *copB* |  | [15] |
| 3474 | *stk* |  | [9] |
| 3502 | *agmI* |  | [9] |
| 3506 | *sgmL* | NAD-dependent epimerase/hydratase; S motility | [9] |
| 3536 | *aglZ* |  | [9] |
| 3537 | *agmL, idh* |  | [9] |
| 3553 |  |  | [50] |
| 3554 |  |  | [50] |
| 3555 |  |  | [50] |
| 3556^d^ |  | hypothetical protein; development | [50] |
| 3596 | *ihfA* | integration host factor; light-induced gene expression | [51] |
| 3692 | *nla18* |  | [18] |
| 3759 | *sgmM, pccB2* |  | [9] |
| 3797 | *sgmN* |  | [9] |
| 3840 | *scpB* |  | [9] |
| 3841 | *scpA* |  | [9] |
| 3842 | *agmD, trpS* |  | [9] |
| 3883 |  |  | [52] |
| 3884 |  |  | [52] |
| 3885 | *sapB* | spore protein | [52, 53] |
| 3886 | *agmA* |  | [9] |
| 3959 |  |  | [4] |
| 3993 | *bsgA* | protease; B-signaling | [54, 55] |
| 4016 | *pfk* |  | [56, 57] |
| 4017 | *pkn4* |  | [56, 57] |
| 4020 | *Mxa213* |  | [3] |
| 4138 | *frzF* |  | [58] |
| 4139 | *frzG* |  | [58] |
| 4140 | *frzE* |  | [58] |
| 4141 | *frzCD* |  | [58] |
| 4142 | *frzB* |  | [58] |
| 4143 | *frzA* |  | [58] |
| 4144 | *frzZ* |  | [58] |
| 4147 | *rpoE1* | ECF σ; A and S motility regulation | [59] |
| 4148 | *pglK* |  | [33] |
| 4149 | *frzS* | RR; S motility | [60] |
| 4150 | *rasA, sgmO* |  | [9, 61] |
| 4196 |  |  | [2] |
| 4261 |  |  | [2] |
| 4263 |  |  | [62] |
| 4289 |  |  | [63] |
| 4290 |  |  | [63] |
| 4291 |  |  | [63] |
| 4292 |  |  | [63] |
| 4293 |  |  | [63] |
| 4294 |  |  | [63] |
| 4295 |  |  | [63] |
| 4296 |  |  | [63] |
| 4297 |  |  | [63] |
| 4298 |  |  | [63] |
| 4299 |  |  | [63] |
| 4300 |  |  | [63] |
| 4301 |  |  | [63] |
| 4302 |  |  | [63] |
| 4303 |  |  | [63] |
| 4304 |  |  | [63] |
| 4305 |  |  | [63] |
| 4333 | *ftsH^D^* | protease that degrades PopD; C-signaling | [6] |
| 4398 | *pph3* |  | [64] |
| 4564 | *esgA* | Branched-chain keto acid dehydrogenase; E-signaling | [65] |
| 4565 | *esgB* |  | [65] |
| 4610 | *rfbC* |  | [9] |
| 4611 | *rfbA* |  | [9] |
| 4613 | *sgmP, rfbB* |  | [9] |
| 4616 | *pglF, sgmQ* |  | [9, 33] |
| 4619 | *wbgB* | glycosyltransferase; S motility | [9] |
| 4621 | *rfbC, rfaC* |  | [9, 66] |
| 4622 | *rfbB, rfaB* |  | [9, 66] |
| 4623 | *rfbA, rfaA* |  | [9, 66] |
| 4638 | *agmH* |  | [9] |
| 4639 | *sgmS* | TPR protein; S motility | [9] |
| 4640 | *sgmT* |  | [9, 67] |
| 4662 | *ecfA* |  | H. Kaplan personal comm. |
| 4707 | *sgmU* |  | [9] |
| 4710 | *pglN, sgnG* |  | [9, 33] |
| 4711 | *lpxK* |  | [9] |
| 4714 | *kdtA* |  | [9] |
| 4751 | *cheY8b* |  | [68] |
| 4752 | *cheB8* |  | [68] |
| 4753 | *cheR8* |  | [68] |
| 4756 | *cheW8b* |  | [68] |
| 4757 | *cheW8a* |  | [68] |
| 4758 | *cheA8* |  | [68] |
| 4759 | *cheY8a* |  | [68] |
| 4761 | *nusB* |  | [68] |
| 4762 | *ribH* |  | [68] |
| 4763 | *ribE* |  | [68] |
| 4764 | *ribD* |  | [68] |
| 4765 | *ybaD* |  | [68] |
| 4787 | *phoP4* |  | [69] |
| 4798 | *agmC* |  | [9] |
| 4832 |  |  | [70] |
| 4862 | *agmX, gltJ* |  | [9, 35] |
| 4863 | *agmK, gltI* |  | [9, 35] |
| 4866 | *agmV, cglE, gltH* | hypothetical protein; A motility | [9, 14, 35] |
| 4867 | *pglI, gltG* |  | [33, 35] |
| 4868 | *cglF, gltF* |  | [14, 35] |
| 4869 | *aglT, gltE* |  | [9, 35] |
| 4870 | *agmU, gltD* |  | [9, 35] |
| 4904 | *MkapB* |  | [27] |
| 5131 | *pdeA* |  | [8] |
| 5144 | *cheR3* |  | [71] |
| 5145 | *cheB3* |  | [71] |
| 5147 | *cheA3* |  | [71] |
| 5148 | *mcp3B* |  | [71] |
| 5149 | *mcp3A* |  | [71] |
| 5150 | *cheW3* |  | [71] |
| 5151 | *crdC* |  | [71] |
| 5184 | *crdS* |  | [72] |
| 5204 | *asgC, rpoD* | σ^A^; gene expression | [73] |
| 5208 | *socA1* | short-chain alcohol dehydrogenase; bypass of C-signaling | [74] |
| 5209 | *socA2* |  | [74] |
| 5210 | *socA3* |  | [74] |
| 5245 |  |  | [4] |
| 5319 | *agiA, pglC* |  | [9, 33] |
| 5327 | *gmd* | GDP-mannose-4,6-dehydratase; S motility | [9] |
| 5328 | *rmd* |  | [9] |
| 5333 | *sgmV* |  | [9] |
| 5364 | *hsfA* |  | [75] |
| 5365 | *hsfB* | HPK; heat-shock gene expression | [75] |
| 5382 | *aspT* |  | [33] |
| 5430 | *tps* |  | [76] |
| 5432 | *ops* |  | [76] |
| 5506 |  |  | [4] |
| 5585 | *pglE* |  | [33] |
| 5592 | *sgmW, digR* |  | [9, 77] |
| 5622 | *carD* |  | [78] |
| 5731 | *ecf* |  | [4] |
| 5744 | *agmW* |  | [9] |
| 5753 | *aglX* |  | [9] |
| 5754 | *aglV* |  | [9] |
| 5756 | *aglW* |  | [9] |
| 5766 | *sgmX* |  | [9] |
| 5769 | *efp* | translation elongation factor P; S motility | [9] |
| 5770 | *sgmY* |  | [9] |
| 5772 | *pilQ* |  | [9, 79] |
| 5773 | *pilP* |  | [9, 79] |
| 5774 | *pilO* |  | [9, 79] |
| 5775 | *pilN* |  | [9, 79] |
| 5776 | *pilM* |  | [9, 79] |
| 5777 | *nla23, pilR1* |  | [18, 36] |
| 5778 | *pilS1* |  | [36] |
| 5779 | *pilD, gspO* |  | [9, 79] |
| 5780 | *pilI* |  | [9, 79] |
| 5781 | *pilH* |  | [9, 79] |
| 5782 | *pilG* |  | [9, 79] |
| 5783 | *pilA* | pilin protein; S motility | [9, 80] |
| 5784 | *pilR* | EBP; S motility | [9, 80] |
| 5785 | *pilS* |  | [9, 80] |
| 5786 | *pilC* |  | [9, 79] |
| 5787 | *pilT* |  | [9, 79] |
| 5788 | *pilB* |  | [9, 79] |
| 5818 | *agmR* |  | [9] |
| 5820 | *agmM* |  | [9] |
| 5831 | *sgmZ, glgP* |  | [9] |
| 5853 | *nla1* |  | [18] |
| 5879 |  |  | [2] |
| 5906 | *mmrA* |  | [9] |
| 5907 | *rppA* |  | [9] |
| 6027 | *mcp5* |  | [68] |
| 6028 | *cheB5* |  | [68] |
| 6029 | *cheA5* |  | [68] |
| 6030 | *cheW5* |  | [68] |
| 6031 | *cheR5* |  | [68] |
| 6032 | *cheV5* |  | [68] |
| 6033 | *cheY5* | RR; signaling | [68] |
| 6106 | *fibA* | protease; lipid signaling | [81, 82] |
| 6125 | *sgnA* | hypothetical protein; S motility | [9] |
| 6259 | *agmJ* | ABC transporter; A motility | [9] |
| 6403 | *agnB* |  | [36] |
| 6501 | *pglD* |  | [33] |
| 6518 | *sgnB* | hypothetical protein; S motility | [9] |
| 6519 | *agmG* | site-specific recombinase; A motility | [9] |
| 6607 | *agmT* |  | [9] |
| 6608 | *agmS* |  | [9] |
| 6627 | *sgnC* |  | [9] |
| 6669 | *pskB7* |  | [10] |
| 6671 | *sglK* |  | [9] |
| 6679 | *sgnH* |  | [9] |
| 6681 |  |  | [4] |
| 6691 | *difG* |  | [83] |
| 6692 | *difE* |  | [83] |
| 6693 | *difD* |  | [83] |
| 6694 | *difC* |  | [83] |
| 6695 | *difB* |  | [83] |
| 6696 | *difA* |  | [83] |
| 6789 | *mreB* |  | [84] |
| 6860 | *aglS* |  | [9, 35] |
| 6861 | *aglQ* |  | [35] |
| 6862 | *aglR* | A motility | [9, 35] |
| 6890 | *hthB* |  | [85] |
| 6908 | *sgnD, pgi* |  | [9] |
| 6947 | *cheW6a* | coupling protein; S motility | [68] |
| 6948 | *cheR6* |  | [68] |
| 6949 | *cheW6b* |  | [68] |
| 6950 | *mcp6* |  | [68] |
| 6951 | *cheA6* |  | [68] |
| 6952 | *cheB6* |  | [68] |
| 6953 | *socD* |  | [68] |
| 6954 | *kefC* |  | [68] |
| 6955 | *todK* |  | [86] |
| 6958 | *des7* |  | [68] |
| 6959 | *cheB7* |  | [68] |
| 6960 | *cheR7* |  | [68] |
| 6961 | *cpc7* |  | [68] |
| 6962 | *mcp7* |  | [68] |
| 6963 | *cheW7* |  | [68] |
| 6964 | *cheA7* |  | [68] |
| 6965 | *cheY7* | RR; S motility and carotenoid synthesis | [68] |
| 7061 | *mbhA* |  | [87] |
| 7068 | *gsmA* |  | [46] |
| 7103 | *sgnI* |  | [9] |
| 7143 |  |  | [2] |
| 7160 | *pglM* |  | [33] |
| 7181 | *MkapA* |  | [27] |
| 7206 | *mokA* | hybrid HPK-RR; signaling | [88] |
| 7252 | *pglA* |  | [33] |
| 7289 |  |  | [4] |
| 7296 | *aglCR* | hypothetical protein; A motility | [36] |
| 7360 | *sgnE* |  | [9] |
| 7370 | *pktF4* |  | [10] |
| 7402 | *nsd* | hypothetical protein; ppGpp-signaling | [89] |
| 7407 | *sapA* | spore protein | [53] |
| 7415 | *epsZ* | sugar transferase; S motility | [90] |
| 7417 | *epsY* |  | [90] |
| 7418 | *epsX* |  | [90] |
| 7420 | *epsW* |  | [90] |
| 7421 | *epsV* |  | [90] |
| 7422 | *epsU* |  | [90] |
| 7426 | *epsT* | hypothetical protein; S motility | [90] |
| 7430 | *epsQ* |  | [90] |
| 7431 | *epsP* |  | [90] |
| 7433 | *epsO* |  | [90] |
| 7435 | *epsN* |  | [90] |
| 7436 | *epsM, czc3C* |  | [90] |
| 7437 | *epsL, czc3A* | heavy metal efflux pump; S motility | [90, 91] |
| 7438 | *epsK, czc3B* | metal resistance protein; S motility | [90, 91] |
| 7439 | *epsJ* | HPK; S motility | [90] |
| 7440 | *nla24, epsI* |  | [18, 90, 92] |
| 7441 | *epsH* |  | [90] |
| 7443 | *epsG, mgtE* |  | [90] |
| 7444 | *epsF* | hybrid RR-HPK; S motility | [90] |
| 7445 | *epsE* |  | [90] |
| 7448 | *epsD* |  | [90] |
| 7449 | *epsC* |  | [90] |
| 7450 | *espB* |  | [90] |
| 7451 | *epsA* |  | [90] |
| 7454 |  |  | [4] |

^a^ Genes that may be in operons are highlighted yellow, or green if likely the first gene of the operon and having a putative MrpC binding site near (i.e., between -400 and +100) their predicted translation start codon (TSC).

^b^ For genes with a putative MrpC binding site near (i.e., between -400 and +100) their predicted TSC, a description of the protein is given, as well as the process in which it is involved. Abbreviations: ABC, ATP-binding cassette; EBP, enhancer binding protein; HPK, histidine protein kinase; HTH, helix-turn-helix motif; NDP, nucleoside diphosphate; RR, response regulator; STPK, serine/threonine protein kinase; TPR, tetratricopeptide repeat.

^c^ *MXAN2912* may be the first gene of this operon and there are two putative MrpC binding sites located at -171 and +279 relative to its predicted TSC (Additional file 3). The latter site is located at -288 relative to the predicted TSC of *MXAN2913*.

^d^ *MXAN3557* may be the first gene of this operon and there is a putative MrpC binding sites located at +214 relative to its predicted TSC (Additional file 3). This site is located at -306 relative to the predicted TSC of *MXAN3556*.

**References**

1. Rosario CJ, Singer M: **Developmental expression of *dnaA* is required for sporulation and timing of fruiting body formation in *Myxococcus xanthus***. *Mol Microbiol* 2010, **76**(5):1322-1333.

2. Giglio KM, Eisenstatt J, Garza AG: **Identification of enhancer binding proteins important for *Myxococcus xanthus* development**. *J Bacteriol* 2010, **192**(1):360-364.

3. Gorski L, Kaiser D: **Targeted mutagenesis of σ^54^ activator proteins in *Myxococcus xanthus***. *J Bacteriol* 1998, **180**(22):5896-5905.

4. Ulrich LE, Zhulin IB: **The MiST2 database: a comprehensive genomics resource on microbial signal transduction**. *Nucleic Acids Res* 2010, **38**(Database issue):D401-D407.

5. Rolbetzki A, Ammon M, Jakovljevic V, Konovalova A, Sogaard-Andersen L: **Regulated secretion of a protease activates intercellular signaling during fruiting body formation in *M. xanthus***. *Dev Cell* 2008, **15**(4):627-634.

6. Konovalova A, Lobach S, Sogaard-Andersen L: **A RelA-dependent two-tiered regulated proteolysis cascade controls synthesis of a contact-dependent intercellular signal in *Myxococcus xanthus***. *Mol Microbiol* 2012, **84**(2):260-275.

7. Furusawa G, Dziewanowska K, Stone H, Settles M, Hartzell P: **Global analysis of phase variation in *Myxococcus xanthus***. *Mol Microbiol* 2011, **81**(3):784-804.

8. Kimura Y, Nakatuma H, Sato N, Ohtani M: **Contribution of the cyclic nucleotide phosphodiesterases PdeA and PdeB to adaptation of *Myxococcus xanthus* cells to osmotic or high-temperature stress**. *J Bacteriol* 2006, **188**(2):823-828.

9. Hartzell P, Shi W, Youderian P: **Gliding motility of *Myxococcus xanthus***. In: *Myxobacteria: Multicellularity and Differentiation.* Edited by Whitworth DE. Washington, DC: ASM Press; 2008: 103-122.

10. Inouye S, Nariya H, Munoz-Dorado J: **Protein Ser/Thr kinases and phosphatases in *Myxococcus xanthus***. In: *Myxobacteria: multicellularity and differentiation.* Edited by Whitworth DE. Washington, DC: ASM Press; 2008: 191-210.

11. Cheng YL, Kalman L, Kaiser D: **The *dsg* gene of *Myxococcus xanthus* encodes a protein similar to translation initiation factor IF3**. *J Bacteriol* 1994, **176**:1427-1433.

12. Kimura Y, Ohtani M, Takegawa K: **An adenylyl cyclase, CyaB, acts as an osmosensor in *Myxococcus xanthus***. *J Bacteriol* 2005, **187**(10):3593-3598.

13. Crawford EW, Shimkets LJ: **The stringent response in *Myxococcus xanthus* is regulated by SocE and the CsgA C-signaling protein**. *Genes Dev* 2000, **14**(4):483-492.

14. Pathak DT, Wall D: **Identification of the *cglC, cglD, cglE,* and *cglF* genes and their role in cell contact-dependent gliding motility in *Myxococcus xanthus***. *J Bacteriol* 2012, **194**(8):1940-1949.

15. Moraleda-Munoz A, Perez J, Extremera AL, Munoz-Dorado J: **Expression and physiological role of three *Myxococcus xanthus* copper-dependent P1B-type ATPases during bacterial growth and development**. *Appl Environ Microbiol* 2010, **76**(18):6077-6084.

16. Keseler I, Kaiser D: **σ^54^, a vital protein for *Myxococcus xanthus***. *Proc Natl Acad Sci USA* 1997, **94**:1979-1984.

17. Hager E, Tse H, Gill RE: **Identification and characterization of *spdR* mutations that bypass the BsgA protease-dependent regulation of developmental gene expression in *Myxococcus xanthus***. *Mol Microbiol* 2001, **39**(3):765-780.

18. Caberoy NB, Welch RD, Jakobsen JS, Slater SC, Garza AG: **Global mutational analysis of NtrC-like activators in *Myxococcus xanthus*: identifying activator mutants defective for motility and fruiting body development**. *J Bacteriol* 2003, **185**(20):6083-6094.

19. Yu YT, Yuan X, Velicer GJ: **Adaptive evolution of an sRNA that controls *Myxococcus* development**. *Science* 2010, **328**(5981):993.

20. Cho K, Treuner-Lange A, O'Connor KA, Zusman DR: **Developmental aggregation of *Myxococcus xanthus* requires *frgA*, an *frz*-related gene**. *J Bacteriol* 2000, **182**(23):6614-6621.

21. Xu D, Yang C, Kaplan HB: ***Myxococcus xanthus sasN* encodes a regulator that prevents developmental gene expression during growth**. *J Bacteriol* 1998, **180**(23):6215-6223.

22. Yang C, Kaplan HB: ***Myxococcus xanthus sasS* encodes a sensor histidine kinase required for early developmental gene expression**. *J Bacteriol* 1997, **179**(24):7759-7767.

23. Ward MJ, Mok KC, Astling DP, Lew H, Zusman DR: **An ABC transporter plays a developmental aggregation role in *Myxococcus xanthus***. *J Bacteriol* 1998, **180**(21):5697-5703.

24. O'Connor KA, Zusman DR: **Genetic analysis of tag mutants of *Myxococcus xanthus* provides evidence for two developmental aggregation systems**. *J Bacteriol* 1990, **172**:3868-3878.

25. Diodati ME, Gill RE, Plamann L, Singer M: **Initiation and early developmental events**. In: *Myxobacteria: Multicellularity and Differentiation.* Edited by Whitworth DE. Washington, D.C.: ASM Press; 2008: 43-76.

26. Martinez-Canamero M, Munoz-Dorado J, Farez-Vidal E, Inouye M, Inouye S: **Oar, a 115-kilodalton membrane protein required for development of *Myxococcus xanthus***. *J Bacteriol* 1993, **175**:4756-4763.

27. Nariya H, Inouye S: **Modulating factors for the Pkn4 kinase cascade in regulating 6-phosphofructokinase in *Myxococcus xanthus***. *Mol Microbiol* 2005, **56**(5):1314-1328.

28. Curtis PD, Geyer R, White DC, Shimkets LJ: **Novel lipids in *Myxococcus xanthus* and their role in chemotaxis**. *Environ Microbiol* 2006, **8**(11):1935-1949.

29. Curtis PD, Shimkets LJ: **Metabolic pathways relevant to predation, signaling, and development**. In: *Myxobacteria: Multicellularity and Differentiation.* Edited by Whitworth DE. Washington, DC: ASM Press; 2008: 241-258.

30. Stephens K, Hartzell P, Kaiser D: **Gliding motility in *Myxococcus xanthus: mgl* locus, RNA, and predicted protein products**. *J Bacteriol* 1989, **171**:819-883.

31. Thomasson B, Link J, Stassinopoulos AG, Burke N, Plamann L, Hartzell PL: **MglA, a small GTPase, interacts with a tyrosine kinase to control type IV pili-mediated motility and development of *Myxococcus xanthus***. *Mol Microbiol* 2002, **46**(5):1399-1413.

32. Treuner-Lange A, Ward MJ, Zusman DR: **Pph1 from *Myxococcus xanthus* is a protein phosphatase involved in vegetative growth and development**. *Mol Microbiol* 2001, **40**(1):126-140.

33. Yu R, Kaiser D: **Gliding motility and polarized slime secretion**. *Mol Microbiol* 2007, **63**(2):454-467.

34. Bragg J, Rajkovic A, Anderson C, Curtis R, Van Houten J, Begres B, Naples C, Snider M, Fraga D, Singer M: **Identification and characterization of a putative arginine kinase homolog from *Myxococcus xanthus* required for fruiting body formation and cell differentiation**. *J Bacteriol* 2012, **194**(10):2668-2676.

35. Luciano J, Agrebi R, Le Gall AV, Wartel M, Fiegna F, Ducret A, Brochier-Armanet C, Mignot T: **Emergence and modular evolution of a novel motility machinery in bacteria**. *PLoS Genet* 2011, **7**(9):e1002268.

36. Kaiser D, Robinson M, Kroos L: **Myxobacteria, polarity, and multicellular morphogenesis**. *Cold Spring Harb Perspect Biol* 2010, **2**(8):a000380.

37. Zhang W, Inouye M, Inouye S: **Reciprocal regulation of the differentiation of *Myxococcus xanthus* by Pkn5 and Pkn6, eukaryotic-like Ser/Thr protein kinases**. *Mol Microbiol* 1996, **20**(2):435-447.

38. Plamann L, Li Y, Cantwell B, Mayor J: **The *Myxococcus xanthus asgA* gene encodes a novel signal transduction protein required for multicellular development**. *J Bacteriol* 1995, **177**:2014-2020.

39. Vlamakis HC, Kirby JR, Zusman DR: **The Che4 pathway of *Myxococcus xanthus* regulates type IV pilus-mediated motility**. *Mol Microbiol* 2004, **52**(6):1799-1811.

40. Kimura Y, Yoshimi M, Takata G: **Enzymatic and mutational analyses of a class II 3',5'-cyclic nucleotide phosphodiesterase, PdeE, from *Myxococcus xanthus***. *J Bacteriol* 2011, **193**(8):2053-2057.

41. Kimura Y, Mishima Y, Nakano H, Takegawa K: **An adenylyl cyclase, CyaA, of *Myxococcus xanthus* functions in signal transduction during osmotic stress**. *J Bacteriol* 2002, **184**(13):3578-3585.

42. Jakobsen JS, Jelsbak L, Welch RD, Cummings C, Goldman B, Stark E, Slater S, Kaiser D: **σ^54^ enhancer binding proteins and *Myxococcus xanthus* fruiting body development**. *J Bacteriol* 2004, **186**(13):4361-4368.

43. Plamann L, Davis J, Cantwell B, Mayor J: **Evidence that *asgB* encodes a DNA-binding protein essential for growth and development of *Myxococcus xanthus***. *J Bacteriol* 1994, **176**:2013-2022.

44. Ueki T, Inouye S: **A new sigma factor, SigD, essential for stationary phase is also required for multicellular differentiation in *Myxococcus xanthus***. *Genes Cells* 1998, **3**:371-385.

45. Cusick JK, Hager E, Gill RE: **Characterization of *bcsA* mutations that bypass two distinct signaling requirements for *Myxococcus xanthus* development**. *J Bacteriol* 2002, **184**(18):5141-5150.

46. Kimura Y, Kawasaki S, Yoshimoto H, Takegawa K: **Glycine betaine biosynthesized from glycine provides an osmolyte for cell growth and spore germination during osmotic stress in *Myxococcus xanthus***. *J Bacteriol* 2010, **192**(5):1467-1470.

47. Muller FD, Schink CW, Hoiczyk E, Cserti E, Higgs PI: **Spore formation in *Myxococcus xanthus* is tied to cytoskeleton functions and polysaccharide spore coat deposition**. *Mol Microbiol* 2012, **83**(3):486-505.

48. Ueki T, Inouye S: **Identification of a gene involved in polysaccharide export as a transcription target of FruA, an essential factor for *Myxococcus xanthus* development**. *J Biol Chem* 2005, **280**(37):32279-32284.

49. Kimura Y, Yamashita S, Mori Y, Kitajima Y, Takegawa K: **A *Myxococcus xanthus* bacterial tyrosine kinase, BtkA, is required for the formation of mature spores**. *J Bacteriol* 2011, **193**(20):5853-5857.

50. Kim D, Chung J, Hyun H, Lee C, Lee K, Cho K: **Operon required for fruiting body development in *Myxococcus xanthus***. *J Microbiol Biotechnol* 2009, **19**(11):1288-1294.

51. Moreno AJ, Fontes M, Murillo FJ: ***ihfA* gene of the bacterium *Myxococcus xanthus* and its role in activation of carotenoid genes by blue light**. *J Bacteriol* 2001, **183**(2):557-569.

52. Leng X, Zhu W, Jin J, Mao X: **Evidence that a chaperone-usher-like pathway of *Myxococcus xanthus* functions in spore coat formation**. *Microbiology* 2011, **157**(Pt 7):1886-1896.

53. Dahl JL, Fordice D: **Small acid-soluble proteins with intrinsic disorder are required for UV resistance in *Myxococcus xanthus* spores**. *J Bacteriol* 2011, **193**(12):3042-3048.

54. Gill RE, Karlok M, Benton D: ***Myxococcus xanthus* encodes an ATP-dependent protease which is required for developmental gene transcription and intercellular signaling**. *J Bacteriol* 1993, **175**:4538-4544.

55. Tojo N, Inouye S, Komano T: **The *lonD* gene is homologous to the *lon* gene encoding an ATP-dependent protease and is essential for the development of *Myxococcus xanthus***. *J Bacteriol* 1993, **175**:4545-4549.

56. Nariya H, Inouye S: **Activation of 6-phosphofructokinase via phosphorylation by Pkn4, a protein Ser/Thr kinase of *Myxococcus xanthus***. *Mol Microbiol* 2002, **46**(5):1353-1366.

57. Nariya H, Inouye S: **An effective sporulation of *Myxococcus xanthus* requires glycogen consumption via Pkn4-activated 6-phosphofructokinase**. *Mol Microbiol* 2003, **49**(2):517-528.

58. McBride MJ, Weinberg RA, Zusman DR: **"Frizzy" aggregation genes of the gliding bacterium *Myxococcus xanthus* show sequence similarities to the chemotaxis genes of enteric bacteria**. *Proc Natl Acad Sci USA* 1989, **86**:424-428.

59. Ward M, Lew H, Treuner-Lange A, Zusman D: **Regulation of motility behavior in *Myxococcus xanthus* may require an extracytoplasmic-function sigma factor**. *J Bacteriol* 1998, **180**:5668-5675.

60. Ward MJ, Lew H, Zusman DR: **Social motility in *Myxococcus xanthus* requires FrzS, a protein with an extensive coiled-coil domain**. *Mol Microbiol* 2000, **37**(6):1357-1371.

61. Pham VD, Shebelut CW, Diodati ME, Bull CT, Singer M: **Mutations affecting predation ability of the soil bacterium *Myxococcus xanthus***. *Microbiology* 2005, **151**(Pt 6):1865-1874.

62. Hao T, Biran D, Velicer GJ, Kroos L: **Identification of the Ω4514 regulatory region, a developmental promoter of *Myxococcus xanthus* that is transcribed *in vitro* by the major vegetative RNA polymerase**. *J Bacteriol* 2002, **184**(12):3348-3359.

63. Meiser P, Bode HB, Muller R: **The unique DKxanthene secondary metabolite family from the myxobacterium *Myxococcus xanthus* is required for developmental sporulation**. *Proc Natl Acad Sci USA* 2006, **103**(50):19128-19133.

64. Kimura Y, Mori Y, Ina Y, Takegawa K: **Enzymatic and functional analysis of a protein phosphatase, Pph3, from *Myxococcus xanthus***. *J Bacteriol* 2011, **193**(10):2657-2661.

65. Toal DR, Clifton S, Roe B, Downard J: **The *esg* locus of *Myxococcus xanthus* encodes the E1*α* and E1β subunits of a branced-chain keto acid dehydrogenase**. *Mol Microbiol* 1995, **16**:177-189.

66. Guo D, Bowden MG, Pershad R, Kaplan HB: **The Myxococcus xanthus rfbABC operon encodes an ATP-binding cassette transporter homolog required for O-antigen biosynthesis and multicellular development**. *J Bacteriol* 1996, **178**(6):1631-1639.

67. Petters T, Zhang X, Nesper J, Treuner-Lange A, Gomez-Santos N, Hoppert M, Jenal U, Sogaard-Andersen L: **The orphan histidine protein kinase SgmT is a c-di-GMP receptor and regulates composition of the extracellular matrix together with the orphan DNA binding response regulator DigR in *Myxococcus xanthus***. *Mol Microbiol* 2012, **84**(1):147-165.

68. Kirby JR, Berleman JE, Muller S, Li D, Scott JC, Wilson JM: **Chemosensory signal transduction systems in *Myxococcus xanthus***. In: *Myxobacteria: Multicellularity and Differentiation.* Edited by Whitworth DE. Washington, DC: ASM Press; 2008: 135-148.

69. Pham VD, Shebelut CW, Jose IR, Hodgson DA, Whitworth DE, Singer M: **The response regulator PhoP4 is required for late developmental events in *Myxococcus xanthus***. *Microbiology* 2006, **152**(Pt 6):1609-1620.

70. Yan J, Garza AG, Bradley MD, Welch RD: **A Clp/Hsp100 chaperone functions in *Myxococcus xanthus* sporulation and self-organization**. *J Bacteriol* 2012, **194**(7):1689-1696.

71. Kirby JR, Zusman DR: **Chemosensory regulation of developmental gene expression in *Myxococcus xanthus***. *Proc Natl Acad Sci USA* 2003, **100**(4):2008-2013.

72. Willett JW, Kirby JR: **CrdS and CrdA comprise a two-component system that is cooperatively regulated by the Che3 chemosensory system in *Myxococcus xanthus***. *MBio* 2011, **2**(4):e00110-00111.

73. Davis J, Mayor J, Plamann L: **A missense mutation in *rpoD* results in an A-signalling defect in *Myxococcus xanthus***. *Mol Microbiol* 1995, **18**:943-952.

74. Lee K, Shimkets L: **Cloning and characterization of the *socA* locus which restores development to *Myxococcus xanthus* C-signaling mutants**. *J Bacteriol* 1994, **176**:2200-2209.

75. Ueki T, Inouye S: **Transcriptional activation of a heat-shock gene, *lonD*, of *Myxococcus xanthus* by a two component histidine-aspartate phosphorelay system**. *J Biol Chem* 2002, **277**(8):6170-6177.

76. Inouye S, Ike Y, Inouye M: **Tandem repeat of the genes for protein S, a development-specific protein of *Myxococcus xanthus***. *J Biol Chem* 1983, **258**:38-40.

77. Overgaard M, Wegener-Feldbrugge S, Sogaard-Andersen L: **The orphan response regulator DigR is required for synthesis of extracellular matrix fibrils in *Myxococcus xanthus***. *J Bacteriol* 2006, **188**(12):4384-4394.

78. Nicolas FJ, Cayuela ML, Martinez-Argudo IM, Ruiz-Vazquez RM, Murillo FJ: **High mobility group I(Y)-like DNA-binding domains on a bacterial transcription factor**. *Proc Natl Acad Sci U S A* 1996, **93**(14):6881-6885.

79. Wall D, Kaiser D: **Type IV pili and cell motility**. *Mol Microbiol* 1999, **32**(1):1-10.

80. Wu SS, Kaiser D: **Genetic and functional evidence that Type IV pili are required for social gliding motility in *Myxococcus xanthus***. *Mol Microbiol* 1995, **18**(3):547-558.

81. Kearns DB, Bonner PJ, Smith DR, Shimkets LJ: **An extracellular matrix-associated zinc metalloprotease is required for dilauroyl phosphatidylethanolamine chemotactic excitation in *Myxococcus xanthus***. *J Bacteriol* 2002, **184**(6):1678-1684.

82. Lee B, Mann P, Grover V, Treuner-Lange A, Kahnt J, Higgs PI: **The *Myxococcus xanthus* spore cuticula protein C is a fragment of FibA, an extracellular metalloprotease produced exclusively in aggregated cells**. *PLoS One* 2011, **6**(12):e28968.

83. Yang Z, Geng Y, Xu D, Kaplan HB, Shi W: **A new set of chemotaxis homologues is essential for *Myxococcus xanthus* social motility**. *Mol Microbiol* 1998, **30**(5):1123-1130.

84. Mauriello EM, Mouhamar F, Nan B, Ducret A, Dai D, Zusman DR, Mignot T: **Bacterial motility complexes require the actin-like protein, MreB and the Ras homologue, MglA**. *Embo J* 2010, **29**(2):315-326.

85. Nielsen M, Rasmussen AA, Ellehauge E, Treuner-Lange A, Sogaard-Andersen L: **HthA, a putative DNA-binding protein, and HthB are important for fruiting body morphogenesis in *Myxococcus xanthus***. *Microbiology* 2004, **150**(Pt 7):2171-2183.

86. Rasmussen AA, Sogaard-Andersen L: **TodK, a putative histidine protein kinase, regulates timing of fruiting body morphogenesis in *Myxococcus xanthus***. *J Bacteriol* 2003, **185**(18):5452-5464.

87. Romeo JM, Zusman DR: **Transcription of the myxobacterial hemagglutinin gene is mediated by a σ^54^-like promoter and a *cis*-acting upstream regulatory region of DNA**. *J Bacteriol* 1991, **173**:2969-2976.

88. Kimura Y, Nakano H, Terasaka H, Takegawa K: ***Myxococcus xanthus mokA* encodes a histidine kinase-response regulator hybrid sensor required for development and osmotic tolerance**. *J Bacteriol* 2001, **183**(4):1140-1146.

89. Brenner M, Garza AG, Singer M: ***nsd*, a locus that affects the *Myxococcus xanthus* cellular response to nutrient concentration**. *J Bacteriol* 2004, **186**(11):3461-3471.

90. Lu A, Cho K, Black WP, Duan XY, Lux R, Yang Z, Kaplan HB, Zusman DR, Shi W: **Exopolysaccharide biosynthesis genes required for social motility in *Myxococcus xanthus***. *Mol Microbiol* 2005, **55**(1):206-220.

91. Moraleda-Munoz A, Perez J, Extremera AL, Munoz-Dorado J: **Differential regulation of six heavy metal efflux systems in the response of *Myxococcus xanthus* to copper**. *Appl Environ Microbiol* 2010, **76**(18):6069-6076.

92. Lancero H, Caberoy NB, Castaneda S, Li Y, Lu A, Dutton D, Duan XY, Kaplan HB, Shi W, Garza AG: **Characterization of a *Myxococcus xanthus* mutant that is defective for adventurous motility and social motility**. *Microbiology* 2004, **150**(Pt 12):4085-4093.
