# Supplementary material for: Transcription factor MrpC binds to promoter regions of hundreds of developmentally-regulated genes in Myxococcus xanthus
Source: BMC Genomics. 2014 Dec 16;15:1123. doi: 10.1186/1471-2164-15-1123 (PMC4320627; doi:10.1186/1471-2164-15-1123)
Supplement: Supplementary file 8 — Additional file 8: Binding of MrpC2 and MrpC to the fruA promoter region. Figure showing a comparison of purified proteins binding to a DNA fragment in EMSAs. (DOCX 47 KB) [file 12864_2014_6823_MOESM8_ESM.docx]

**Additional file 8 Binding of MrpC2 and MrpC to the *fruA* promoter region.** EMSAs with ^32^P-labeled *fruA* DNA (2 nM) spanning from positions -185 to -41 relative to the transcription start site and no protein (-) or a 2-fold dilution series of His_10_-MrpC2 or His_10_-MrpC beginning at 0.5 μM.
