# Supplementary material for: Transcription factor MrpC binds to promoter regions of hundreds of developmentally-regulated genes in Myxococcus xanthus
Source: BMC Genomics. 2014 Dec 16;15:1123. doi: 10.1186/1471-2164-15-1123 (PMC4320627; doi:10.1186/1471-2164-15-1123)
Supplement: Supplementary file 9 — Additional file 9: Summary of His 10 -MrpC2 and FruA-His 6 binding to DNA fragments in EMSAs. List of putative MrpC binding sites tested for binding of MrpC2 and FruA, including peak rank, gene number and name (if one has been assigned), position of the peak coordinate relative to the nearest predicted TSC, relevant characteristics, the apparent number of MrpC2 binding sites in EMSAs with fragments generated by PCR, the number of motifs bound in EMSAs with annealed oligonucleotides, whether FruA binding was observed in EMSAs with fragments generated by PCR, and whether there was evidence of cooperative binding of FruA and MrpC2. (DOCX 14 KB) [file 12864_2014_6823_MOESM9_ESM.docx]

| Rank^a^ | MXAN | Gene | Position of peak relative to ORF start^a^ | Characteristics^b^ | Apparent number of His_10_-MrpC2 binding sites^c^ | Motifs bound by His_10_-MrpC2^d^ | Binding by FruA-His_6_ alone^e^ | Cooperative binding by His_10_-MrpC2 and FruA-His_6_^e^ |
| --- | --- | --- | --- | --- | --- | --- | --- | --- |
| 1 | 4360 |  | 568 | high rank | 2 | 1 | + | + |
| 2 | 6247 |  | -71 | high rank, down-regulated | 2 | 1 | + | + |
| 3 | 4064 |  | -241 | high rank | Nt | 1 | Nt | Nt |
| 4 | 5802 |  | -370 | high rank | 3 | 1 | - | + |
| 5 | 0524 |  | -97 | high rank, up-regulated | 2 | 2 | + | + |
| 6 | 5125 | *mrpC* | -247 | high rank, up-regulated, known to be bound by MrpC/2 and involved in development | >4 | Nt | - | + |
| 12 | 4147 | *rpoE1* | -23 | A and S motility regulation | 4 | Nm | - | + |
| 14 | 3117 | *fruA* | -330 | up-regulated, known to be bound by MrpC/2 and involved in development | 2 | 1 | Nt | Nt |
| 19 | 3993 | *bsgA* | -47 | weakly up-regulated, known to be involved in development | 2 | 1 | + | + |
| 27 | 1710 | *pkn8* | -204 | up-regulated, known to be involved in development | 1 | 1 | - | + |
| 50 | 6947 | *cheW6a* | -55 | S motility | 1 | 1 | + | + |
| 268 | 5208 | *socA1* | -61 | bypass of C-signaling | 2 | Nm | - | - |
| 383 | 6500 | *pktA1* | -74 | known to be involved in development | 2 | 1 | + | + |
| 421 | 4149 | *frzS* | -163 | S motility | 1 | Nm | - | + |
| 444 | 2902 | *Mx3320* | -40 | up-regulated, known to be involved in development | 1 | Nm | + | + |
| 461 | 5123 | *mrpA* | -171 | up-regulated, known to be involved in development | 2 | Nm | + | + |
| 655 | 5783 | *pilA* | -173 | S motility | 1 | Nm | - | - |

**Additional file 9 Summary of His_10_-MrpC2 and FruA-His_6_ binding to DNA fragments in EMSAs**

**^a^** From Additional file 3. Note that the ChIP-seq peak at -330 relative to the *fruA* start codon is at -98 relative to the predicted start codon of MXAN_3116, as listed in Additional file 3.

^b^ From Additional files 3, 6, and 7. See the text or Additional file 6 for references.

^c^ From Figure 4 and Additional file 10. Nt, not tested.

^d^ From Figure 5. Nt, not tested; Nm, no match.

^e^ From Figure 6 and Additional file 10. Nt, not tested. Note that cooperative binding was not observed at a low concentration of His_10_-MrpC2 for MXAN_5802.
