# Supplementary material for: Transcription factor MrpC binds to promoter regions of hundreds of developmentally-regulated genes in Myxococcus xanthus
Source: BMC Genomics. 2014 Dec 16;15:1123. doi: 10.1186/1471-2164-15-1123 (PMC4320627; doi:10.1186/1471-2164-15-1123)
Supplement: Supplementary file 10 — Additional file 10: Patterns of MrpC2 and FruA binding to candidate genes from ChIP-seq. Figure showing EMSAs of purified proteins binding to DNA fragments generated by PCR. (DOCX 429 KB) [file 12864_2014_6823_MOESM10_ESM.docx]

**Additional file 10 Patterns of MrpC2 and FruA binding to candidate genes from ChIP-seq.** For the indicated genes, approximately 200 bp of DNA surrounding a peak from the ChIP-seq analysis was amplified by PCR with ^32^P-labeled primers. These DNA probes (2 nM) were incubated with His_10_-MrpC2 (1 μM) and/or FruA-His_6_ (3 μM) as indicated (unless noted below), and subjected to EMSAs. A lower concentration of His_10_-MrpC2 (0.1 μM in lanes 22 and 24; 0.03 μM in lanes 33 and 34) and FruA-His_6_ (1.5 μM in lanes 23 and 24) was used in some experiments. Filled arrowheads pointing leftward or rightward are inferred to indicate complexes with one or more FruA-His_6_ bound, respectively. Brackets and open arrowheads indicate novel or more abundant complexes produced by the combination of proteins than by either protein alone. The asterisk denotes a complex in lane 10 inferred to have two His_10_-MrpC2 bound, based on its migration. Panels with a narrower separation were analyzed on the same gel, with intervening lanes removed.
