# Supplementary material for: Transcription factor MrpC binds to promoter regions of hundreds of developmentally-regulated genes in Myxococcus xanthus
Source: BMC Genomics. 2014 Dec 16;15:1123. doi: 10.1186/1471-2164-15-1123 (PMC4320627; doi:10.1186/1471-2164-15-1123)
Supplement: Supplementary file 11 — Additional file 11: Roles of genes implicated to be under direct control of MrpC in the signaling and gene regulatory network during M. xanthus development. Diagrams depicting roles of putative MrpC-controlled genes in the MrpC module of the network and in the overall network. (DOCX 39 KB) [file 12864_2014_6823_MOESM11_ESM.docx]

**A**

**B**

**Additional file 11 Roles of genes implicated to be under direct control of MrpC in the signaling and gene regulatory network during *M. xanthus* development.** (A) Roles of genes implicated to be up-regulated (green) or down-regulated (red) in the overall network. See Additional file 1 for an explanation of the network. Two green arrows have been added to depict positive regulation of ppGpp-signaling by CsgA and of *exoE-I* expression by FruA*. Red lines indicate negative regulation of ppGpp-signaling by SocE and Nsd. (B) Feedback loops in the MrpC module. Protein products of genes implicated to be up-regulated by MrpC are shown with green lettering. Red lines indicate potential negative feedback loops that would diminish MrpC activity. Green arrows indicate the same positive feedback loops as in Additional file 2, which shows a more complete diagram of the MrpC module.
