# Supplementary material for: Transcription factor MrpC binds to promoter regions of hundreds of developmentally-regulated genes in Myxococcus xanthus
Source: BMC Genomics. 2014 Dec 16;15:1123. doi: 10.1186/1471-2164-15-1123 (PMC4320627; doi:10.1186/1471-2164-15-1123)
Supplement: Supplementary file 12 — Additional file 12: Primers for PCR and oligonucleotides for EMSAs. The list of primers used to generate DNA fragments by PCR and the list of oligonucleotides that were annealed to produce probes for EMSAs. (DOCX 13 KB) [file 12864_2014_6823_MOESM12_ESM.docx]

**Additional file 5 Primers for PCR and oligonucleotides for EMSAs**

**Primers**

| MXAN | Sequence |
| --- | --- |
| 4360 | CCTCCACCGCGAAGCCAG  GTGGGCATCCACGAAGCGC |
| 6247 | GAGCAATACGGCGCACGG  CCAGGGAACGTAGAAGTCTG |
| 5802 | GCTTCTTCCACCTCATATACATGGTCC  CGAGCTCCTGTGAACCGAAG |
| 0524 | ATCGAGGAGTCCGCAGGCAC  CGTTGAACAATCTATACACGC |
| 5125 | CTGCTCCGACACTCATGCCC  GGCGAACGGGACTTTCCTTC |
| 4147 | GGAATATGTTTCCCTGGCCG  GAATAGAGCGGGTCCAGATG |
| 3117 | GTTGACAGACAGCGCCGTG  CCCCAATGGGAGTGTCCAG |
| 3993 | GACTTCCGAGGAACCCTGG  GTCTTCCTTGTTGATGAGCC |
| 1710 | CTCTTTGCCATCGATTGCAG  GGATTTGCCTTTGCACCTCC |
| 6947 | GCACAGCGAGCACCTGTC  GTACCAACTCGCGCACGTC |
| 5208 | GCTTCTTCCTGGTCGCTGC  CCTCTACTTTGGAACCGGTC |
| 6500 | GTCCACGTCACACCGCATG  GTGCGCTGGAGAAACGCC |
| 4149 | CGAGCCGCTGTATTCGCTC  CAAGGTGATGATGTGCGTCG |
| 2902 | GCTCGTTTGCTTGTTGATACC  CCGTCTCACTGATGTTGACG |
| 5123 | GAAGACCAGCGCGTACAGG  CTCGTTTACGAACTCGTCTG |
| 5783 | GATTAGCACTACGCATGCCAAC  CTTTCGTTCCGTTCATTCCGC |

**Oligonucleotides**

| MXAN | Sequence^a^ |
| --- | --- |
| 4360 | GTTCATCGGGTCTGTCACATAGGCGACACCCTGCTCCGC  GCGGAGCAGGGTGTCGCCTATGTGACAGACCCGATGAAC |
| 6247 | CACTTGGATAAGTGTTCAGCGCCTAACTATAACGCCCCC  GGGGGCGTTATAGTTAGGCGCTGAACACTTATCCAAGTG |
| 4064 | CATCGCGGCTGTGTTCACTACGTGTCGGAGTATGAGTGG  CCACTCATACTCCGACACGTAGTGAACACAGCCGCGATG |
| 5802 | GCGAAGCAGTCTGTCGGCTACTTGACCGTGCTTGCCAGT  ACTGGCAAGCACGGTCAAGTAGCCGACAGACTGCTTCGC |
| 0524 A | CCGTCCCAACAACTGATACACAGTCAACGTTTCTCCATCC  GGATGGAGAAACGTTGACTGTGTATCAGTTGTTGGGACGG |
| 0524 B | CACAGTCAACGTTTCTCCATCCAGCCCACCTGTCCTCCAC  GTGGAGGACAGGTGGGCTGGATGGAGAAACGTTGACTGTG |
| 524 C | CATCCAGCCCACCTGTCCTCCACTGAACACGTACTGGCAC  GTGCCAGTACGTGTTCAGTGGAGGACAGGTGGGCTGGATG |
| 3117 | GACGCCCGGGCGTGTCCGGCTCCGGACAGCGATGACGAA  TTCGTCATCGCTGTCCGGAGCCGGACACGCCCGGGCGTC |
| 3993 | AAACCTGGAAATGTTCACTGGTTGTCGAAGAGAAAAGGC  GCCTTTTCTCTTCGACAACCAGTGAACATTTCCAGGTTT |
| 1710 | GCGGTTCACTGTGTTTGCTAAGGGACAATCAAGGCCGTG  CACGGCCTTGATTGTCCCTTAGCAAACACAGTGAACCGC |
| 6947 | GGCCGACATCAATGTTCGACAGTGGCCACGCCCACGTCT  AGACGTGGGCGTGGCCACTGTCGAACATTGATGTCGGCC |
| 6500 | CAGAGGCCCCTTGTCCACTCATCAACAGAGCAAGCCCCC  GGGGGCTTGCTCTGTTGATGAGTGGACAAGGGGCCTCTG |

^a^ The sequence matching the motif for MrpC binding (TGTYN_8_RAC) is highlighted yellow in one oligonucleotide of each pair. In some cases, there is one mismatch to the motif.
